# PINOID regulates floral organ development by modulating auxin transport and interacts with MADS16 in rice

**DOI:** 10.1111/pbi.13340

**Published:** 2020-02-04

**Authors:** Hua‐Mao Wu, Dong‐Jiang Xie, Zuo‐Shun Tang, Dong‐Qiao Shi, Wei‐Cai Yang

**Affiliations:** ^1^ State Key Laboratory of Molecular Developmental Biology Institute of Genetics and Developmental Biology Chinese Academy of Sciences Beijing China; ^2^ College of Advanced Agricultural Sciences University of Chinese Academy of Sciences Beijing China

**Keywords:** PINOID, MADS16, floral organ development, auxin, rice (*Oryza sativa* L.)

## Abstract

In rice (*Oryza sativa* L.), floral organ development is an important trait. Although a role for *PINOID* in regulating floral organ development was reported recently, the underlying molecular mechanism remains unclear. Here, we isolated and characterized an abnormal floral organ mutant and mapped the causative gene through an improved MutMap method. Molecular study revealed that the observed phenotype is caused by a point mutation in *OsPINOID* (*OsPID*) gene; therefore, we named the mutation as *ospid‐4*. Our data demonstrate that OsPID interacts with OsPIN1a and OsPIN1b to regulate polar auxin transport as shown previously. Additionally, OsPID also interacts with OsMADS16 to regulate transcription during floral organ development in rice. Together, we propose a model that OsPID regulates floral organ development by modulating auxin polar transport and interaction with OsMADS16 and/or LAX1 in rice. These results provide a novel insight into the role of *OsPID* in regulating floral organ development of rice, especially in stigma development, which would be useful for genetic improvement of high‐yield breeding of rice.

## Introduction

Flowers are complex organs which ensure successful reproduction for angiosperm plants. A flower is typically composed of four types of organs arranged in four whorls, namely from the outside to inside, the sepal, the petal, the stamen and the carpel. In rice, a flower is composed of two hulls (lemma and palea), two lodicules, six stamens and a pistil. The stamen consists of filament and anther with four pollen sacs, and the pistil is composed of an ovary/carpel and bifurcates just above the ovary to produce two styles and stigmas covered with papillae cells (Ciampolini *et al.*, 2001; Guo *et al.*, 2015; Yoshida and Nagato, 2011). According to the initial order of floral organ primordia, rice spikelet development can be divided into eight stages (Sp1–Sp8), the primordia of lemma, palea, lodicule, stamen and carpel are formed at Sp3, Sp4, Sp5, Sp6 and Sp7, respectively, and two style primordia are generated at Sp8 (Ikeda *et al.*, 2004).

The floral organs are genetically controlled by transcription factors as formulated by the ‘ABC model’ initially and expanded to ‘ABCD model,’ ‘ABCDE model’ and most recently quartet model (Bowman *et al.*, 1991; Coen and Meyerowitz, 1991; Guo *et al.*, 2015). Even though these models can explain the development of most flowers, the molecular mechanism of floral organ development is complicated. There are also A, B, C, D and E‐class genes in rice. *OsMADS16/SPW1* (*SUPERWOMAN1*) is homologous to *AP3* of *Arabidopsis* (*Arabidopsis thaliana* L.) and mainly expressed in the lodicules and stamens, and also in the pistil. In the *osmads16* mutant, the lodicules develop into a palea‐like structure, and the stamens develop into carpels (Moon *et al.*, 1999; Nagasawa *et al.*, 2003). OsMADS16/SPW1 regulates stamen and carpel identities by acting synergistically and antagonistically with DL (DROOPING LEAF) (Nagasawa *et al.*, 2003). In addition, OsMADS16/SPW1 regulates the morphology of floral organs through interaction with OsMADS3 and OsMADS58 (Yun *et al.*, 2013). These results indicate that OsMADS16/SPW1 mainly regulates the development of the lodicules and stamens, and is also involved in the regulation of carpel identity. In the RNAi‐*OsMADS58* plants, the flower consists of repetitively formed lodicules, stamens and carpel‐like organs, indicating that the determinacy of the floral meristem is severely destroyed. Therefore, OsMADS58 most likely controls the development of carpels and the determinacy of the flower meristem (Yamaguchi *et al.*, 2006).

The development of plant floral organs not only requires genes regulating their identities, but also genes controlling floral meristem size and subsequently the number of floral organs. In *Arabidopsis*, floral meristem size and organ numbers are controlled by a feedback loop driven by WUS and the signalling cascade mediated by the CLAVATA3 (CLV3) glycopeptide and its receptors CLV1, CLV2, CRN and TOAD2 (Brand *et al.*, 2000). Similarly in rice, floral organ numbers are controlled by *FLORAL ORGAN NUMBER* (*FON*) *1* and *FON2/4*‐medicated signalling cascade. *FON1* encodes a leucine‐rich repeat receptor kinase homologous to *CLV1*. In *fon1‐2* plants, 70% of florets have 4–5 pistils and 7–12 stamens (Suzaki *et al.*, 2004). *FON2/4* encodes the CLE peptide homologous to CLV3 of *Arabidopsis*, and the number of pistils in the *fon2‐1*, *fon2‐2* and *fon2‐3* mutants increase by 2.9‐fold, 2.2‐fold and twofold, respectively (Chu *et al.*, 2006; Suzaki *et al.*, 2006). In addition, accumulating evidences suggest that auxin plays a critical role in the regulation of inflorescence, floral meristem and floral organ development (McSteen, 2010; Smyth, 2018; Zhao, 2018). *TRYPTOPHAN DEFICIENT DWARF 1* (*OsTDD1*) (Sazuka *et al.*, 2009), *OsIAA20* (Yoshida *et al.*, 2012), *OsARF6*, *OsAUX/IAA* and *DIOXYGENASE FOR AUXIN OXIDATION* (*DAO*; Zhao *et al.*, 2013) have been shown to play a role in regulating floral organ number and development. Furthermore, plants overexpressing *OsPID* produce more than two pistils and fewer than six stamens (Morita and Kyozuka, 2007). Although recent two studies have shown that loss of *OsPID* function leads to aberrant pistil and anther development (He *et al.*, 2019; Xu *et al.*, 2019), molecular mechanism of *PID* in regulating rice floral organs remains elusive.

Here, we isolated and characterized an abnormal floral organ mutant that produces excessive number of curled anthers and pistils with aberrant style and stigma, or without a style. The gene responsible for the phenotype was identified as *OsPID* using an improved MutMap method and further confirmed by genetic complementation and the clustered regularly interspaced short palindromic repeat (CRISPR)/Cas9‐edited lines. The mutant phenotype is caused by a single nucleotide nonsynonymous mutation in *OsPID* gene, designated as *ospid‐4*. Subcellular localization showed that OsPID is localized to the plasma membrane, nucleus and cytoplasm. Furthermore, we showed that OsPID interacts with OsPIN1a and OsPIN1b, as well as OsMADS16. Taking together, we propose that OsPID regulates floral organ development by modulating auxin polar transport and interaction with OsMADS16 and/or LAX1 in rice. These data will enrich our understanding of the molecular mechanisms of floral organ development and provide useful information for genetic improvement of high‐yield breeding of rice.

## Results

### Identification of an abnormal floral organ mutant

**Figure 1:**
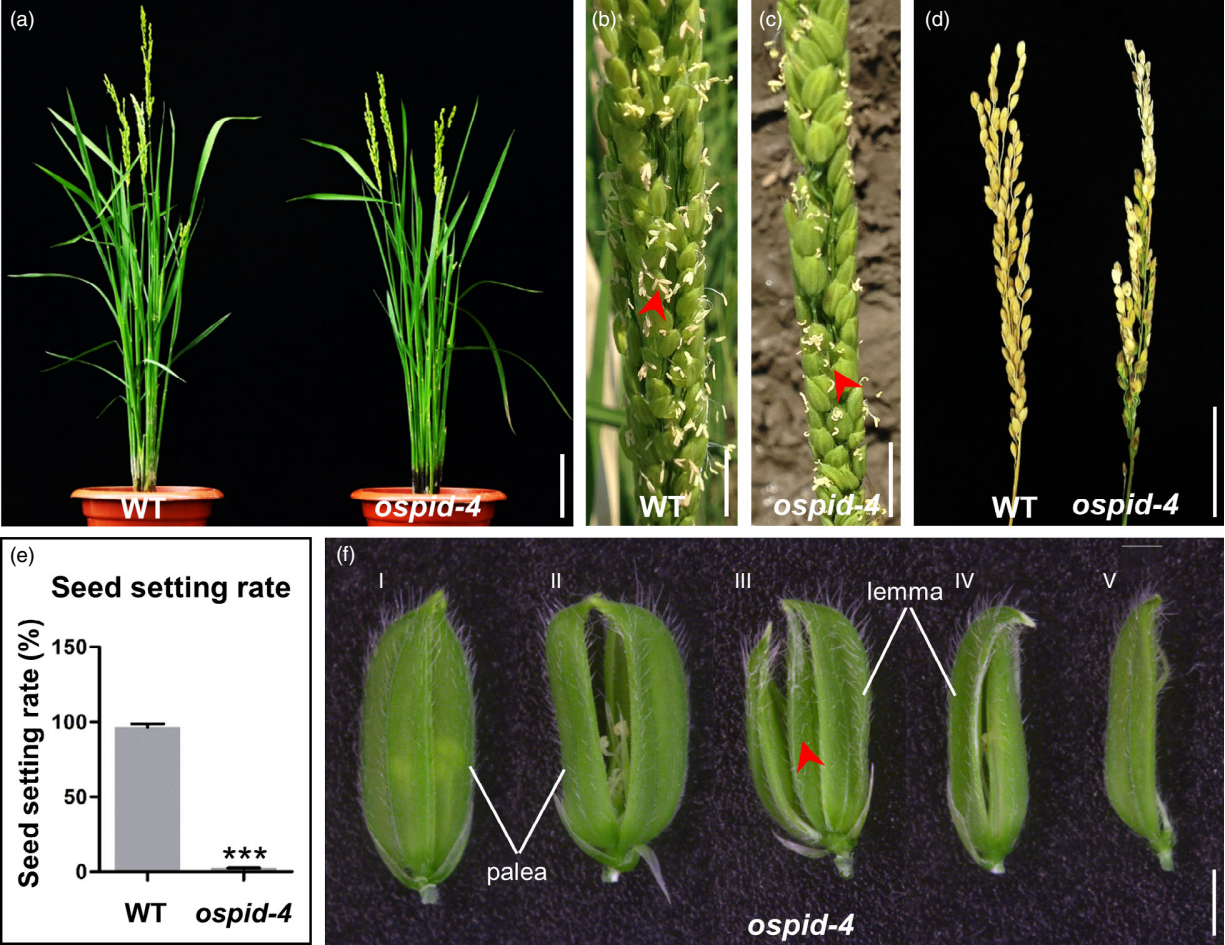
Identification of the *ospid‐4* mutant. (a) Morphological comparison of WT and *ospid‐4* before flowering. Bar, 10 cm. (b,c) WT (b) and *ospid‐4* (c) in flowering stage. Red arrows indicate normal (b) and curved (c) stamens. Bars, 1 cm. (d) Comparison of seed set of WT and *ospid‐4*. Bar, 5 cm. (e) Seed setting rate of WT and *ospid‐4*. Values are means ± SD (*n* = 5), and asterisks represent significant difference between WT and *ospid‐4* (***P < 0.001). (f) Hull types of *ospid‐4*. (I) Normal hull; (II) Palea and lemma cannot be completely closed, and their apex is hooked; (III) Lodicules inside the lemma change to a pair of palea and lemma; (IV) Hull is smaller than WT, the apex of lemma is hooked, and the palea with abnormal development is smaller than the normal lemma; (V) Palea development is severely affected. Red arrow indicates an extra pair of palea and lemma. Bars, 2 mm.

A mutant with abnormal floral organs (designated as *ospid‐4*, see below) was obtained from an ethyl methane sulphonate (EMS) mutant library. During the vegetative development, no morphological differences were observed between the mutant and the wild‐type (WT) plants (Figure 1a). After flowering, however, fewer anthers of the mutant flowers grew out of the lemma and palea compared with the WT (Figure 1b,c). At maturity, the seed setting rate of the mutant was severely affected with only 2.2% compared with 96.4% in WT (Figure 1d,e). Before flowering, the lemma and palea of WT florets grow normally and are completely closed, while there were five types of hulls in *ospid‐4* florets. Type I (75.6%, *n* = 406), lemma and palea are completely closed, just like WT; Type II (17.2%, *n* = 406), palea and lemma cannot be completely closed, and their apex is hooked; Type III (1.2%, *n* = 406), lodicules inside the lemma change to a pair of palea and lemma; Type IV (4%, *n* = 406), hull is smaller than WT, the apex of lemma is hooked, and the palea is smaller than the normal lemma; Type V (2%, *n* = 406), the palea development is severely affected (Figure 1f; Table S1). In total, 24.4% of *ospid‐4* florets have abnormal lemma and palea or lodicules, which indicates that whorl 1 and whorl 2 organs of *ospid‐4* mutant are developed abnormally. In the F_2_ segregating population, the progeny segregated in a 164 : 44 ratio for plants with normal floret and mutant floret, respectively, conforming to a segregation ratio of 3 : 1 (Table S2). This suggests that the phenotype of the mutant is caused by a single recessive mutation.

### Mutation of *OsPID* affects stamen development

**Figure 2:**
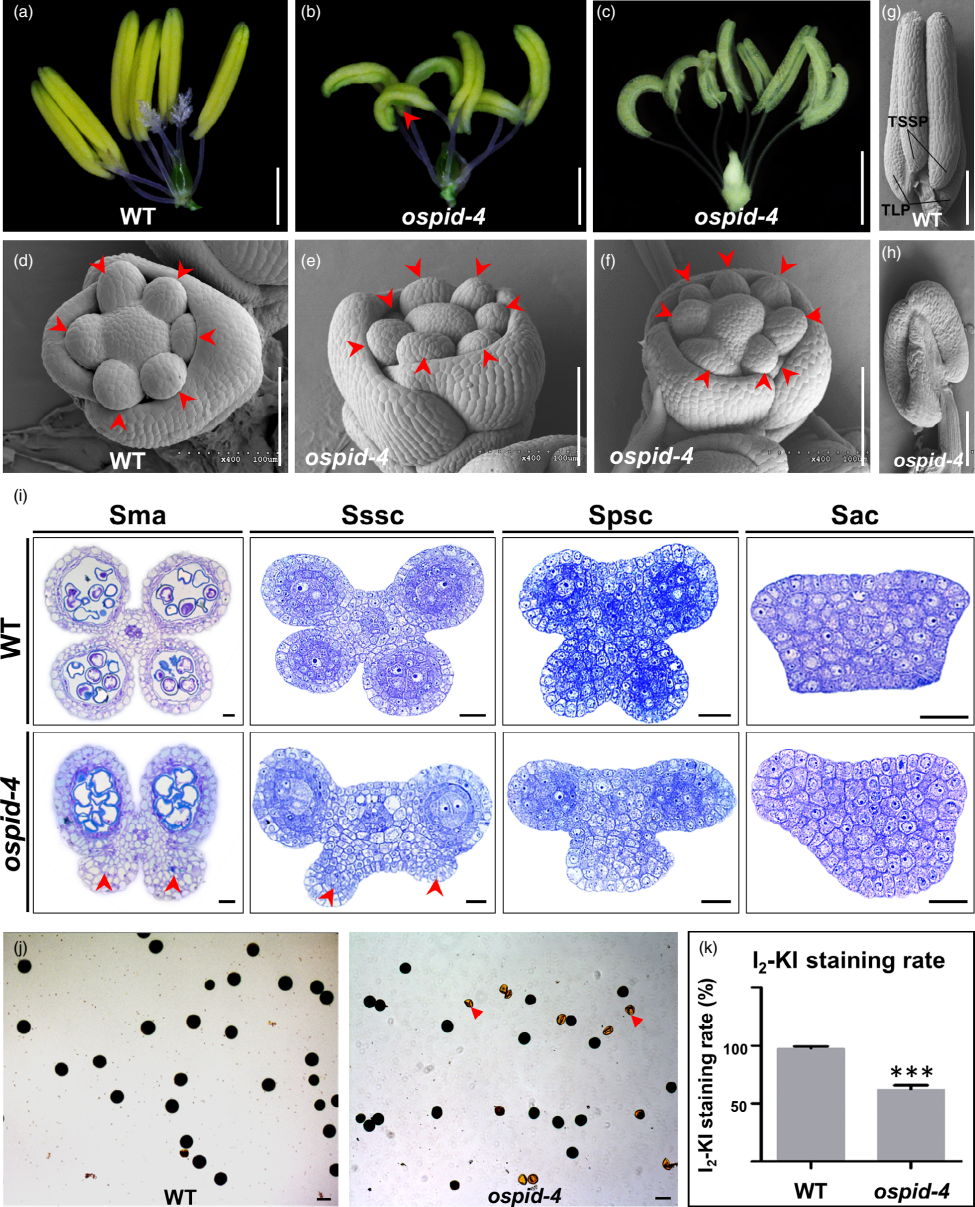
Phenotypic characterization of *ospid‐4* stamens. (a–c) Comparison of pistil and stamen between WT and *ospid‐4*. The normal pistil with double stigmas and plenty stigma hair and six stamens in WT (a), the pistil without stigma and six (b) and eight (c) curved stamens in *ospid‐4*. Red arrow indicates curved stamen. Bars, 1 cm. (d–f) SEM images of stamen primordia of WT and *ospid‐4*. (d) WT floret with six stamen primordia, (e,f) *ospid‐4* florets with seven (e) or eight (f) stamen primordia. Red arrows show stamen primordia. Bars, 100 μm. (g,h) SEM images of young anthers from WT and *ospid‐4*. TLP and TSSP (g) in young anthers of WT, and abnormal pollen sacs (h) in young anthers of *ospid‐4*. Red arrow indicates dysplastic pollen sacs. Bars, 100 μm. (i) Transverse sections of anthers from WT (top) and *ospid‐4* (bottom). Red arrows indicate dysplastic pollen sacs. Sma, Stage of mature anther; Sssc, Stage of the secondary sporogenous cell formation; Spsc, Stage of the primary sporogenous cell formation; Sac, Stage of archesporial cell formation. Bars, 20 μm. (j) I_2_‐KI staining assay of WT and *ospid‐4* pollen. Red arrows indicate sterile and shrivelled pollen. Bars, 50 μm. (k) The I_2_‐KI staining rate of WT and *ospid‐4* pollen. Values are means ± SD (*n* = 3), and asterisks represent significant difference between WT and *ospid‐4* (***P < 0.001).

To investigate floral phenotypes, the lemma and palea of *ospid‐4* mutant were peeled off and analysed. The data showed that the anthers are curled with abnormal pollen sacs, and their numbers vary from six to nine (Figure 2a–c). 14.1% (*n* = 603, Table S3) of *ospid‐4* florets have more than six stamens (Figure 2c) as that in WT florets (Figure 2a). Scanning electron microscopy (SEM) showed that six normal stamen primordia were formed and evenly arranged in WT (Figure 2d), while seven or eight stamen primordia were unevenly arranged in *ospid‐4* florets (Figure 2e,f). This indicated that the number of stamen is caused by increased stamen primordia early in flower development.

Each anther has four normal pollen sacs, two long pollen sacs (TLP) and two slightly short pollen sacs (TSSP) in WT (Figure 2g). While the curved anthers of *ospid‐4* mutant often contain abnormal TLP and TSSP (Figure 2h), the pollen sacs of *ospid‐4* mutant can be divided into five types (Figure S1a). 0.03% (*n* = 3692, Table S4) of *ospid‐4* stamens (Type I) had TLP and TSSP, as WT stamens; 5.47% (*n* = 3692, Table S4) of *ospid‐4* stamens (Type II) had TLP and two short pollen sac; 18.01% (*n* = 3692, Table S4) of *ospid‐4* stamens (Type III) had TLP and one short pollen sac; 75.30% (*n* = 3692, Table S4) of *ospid‐4* stamens (Type IV) had only TLP; 1.19% (*n* = 3692, Table S4) of two adhesive anthers (Type V) were observed in *ospid‐4* florets. Overall, more than 99% of the mutant anthers were abnormal in pollen sacs, and Type III accounts for 75.30%.

To better understand how the developmental defects of the pollen sacs are formed, we traced the developmental progress of the anthers of WT and *ospid‐4* mutant by semi‐thin sectioning. Typically, a mature anther contains four normal pollen sacs in the WT, whereas in *ospid‐4* mutant, two long and two developmentally stagnant pollen sacs were observed (Figure 2i). This morphological variation can be traced back to anther primordial stage when archesporial cell formation is initiated. In WT, anther primordia are oval or rectangular shaped with archesporial cell differentiation at four corners which develop into the typical anther with four pollen sacs (Figure 2i; Zhang *et al.*, 2011a). In *ospid‐4* mutant, however, most of the anther primordia were not quadrangular prismatic and often deformed, and some of them were trapezoidal (Figure 2i). Therefore, anthers with variable pollen sacs were produced. These microscopic analysis shows that the deformed anther development is caused by abnormal anther primordia (Figure 2i), indicating the gene is required in early anther primordium formation.

To further check whether pollen in the mutant anthers are viable, pollen grains were stained with 1% (w/v) iodine‐potassium iodide (I_2_‐KI). The result showed that 62.1% (*n* = 593) of *ospid‐4* pollen grains were viable compared with 97.6% (*n* = 592) of WT (Figure 2j,k). This indicates that the pollen fertility is decreased in *ospid‐4*.

### Pistil development is impaired in *ospid‐4*

**Figure 3:**
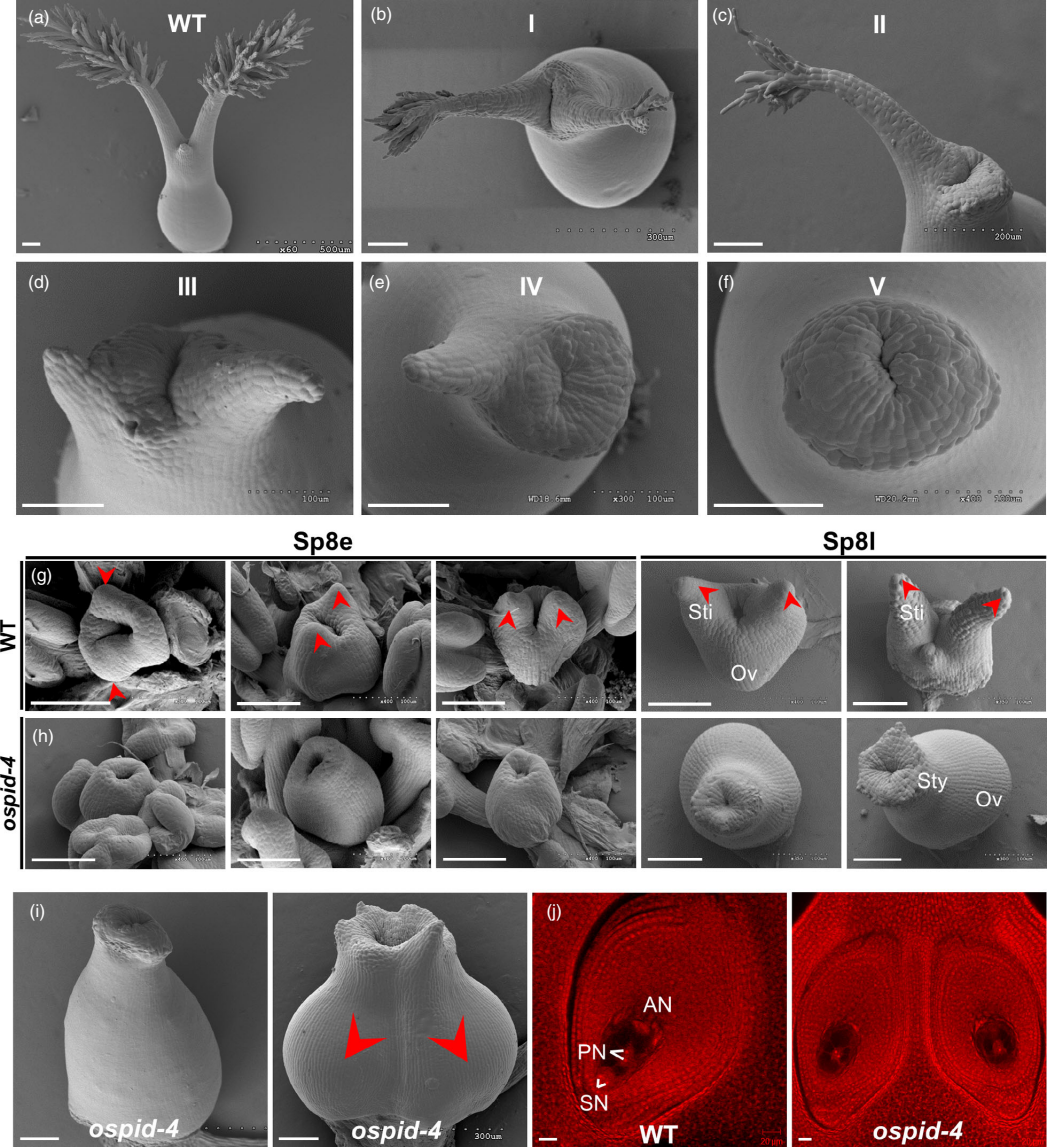
Phenotypic characterization of *ospid‐4* pistil. (a–f) SEM images of pistil from WT and *ospid‐4*. WT pistil had double stigmas with more stigma hair (a); *ospid‐4* pistil had double stigmas with less stigma hair (type I) (b); *ospid‐4* pistil had single stigma with less stigma hair (type II) (c); *ospid‐4* pistil had short‐double styles without stigma (type III) (d); *ospid‐4* pistil had short‐single style without stigma (type IV) (e); *ospid‐4* pistil had no style (type V) (f). Bars, 100 μm. (g,h) SEM images of stigma primordium development in WT (g) and *ospid‐4* (h) at early Sp8 (Sp8e) and later Sp8 (Sp8l). Ov, Ovary; Sti, Stigma; Sty, Style. Red arrows show stigma primordium or stigma. Bars, 100 μm. (i) SEM images of single ovule (left) and double ovules (right) in *ospid‐4* pistil. Red arrows show double ovules. Bars, 100 μm. (j) Comparison of ovules from WT (left) and *ospid‐4* (right). AN, antipodal cell nucleus; PN, polar nucleus; SN, synergid nucleus. Bars, 20 μm.

In addition to abnormal anther, pistil development is also affected in *ospid‐4* mutant. In WT flower, each pistil has a bifurcated style and hairy stigma (Figure 3a), while in *ospid‐4* mutant the number of style and stigma varies or no style at all (Figure 3b–f). Five kinds of abnormal pistil were observed in *ospid‐4* florets. The first type of *ospid‐4* pistil (16.7%, *n* = 603; Table S5) had double stigmas with less stigma hair (Figure 3b); the second type (6.8%, *n* = 603; Table S5) had single stigma with less stigma hair (Figure 3c); and the third type (25.6%, *n* = 603; Table S5) had short‐double styles without stigma (Figure 3d); the fourth type of *ospid‐4* pistil (26.6%, *n* = 603; Table S5) had short‐single style without stigma or hair (Figure 3e); the last type of *ospid‐4* pistil (24.0%, *n* = 603; Table S5) had no style (Figure 3f). Therefore, all the *ospid‐4* pistil were abnormally developed, of which 83% lacking two complete stigmas and 76% of *ospid‐4* pistil without stigma, indicating that the pistil development of the *ospid‐4* mutant is severely affected by the mutation.

To trace when the developmental defects occur, we compared the stigma development of WT and *ospid‐4* mutant at different stages by SEM. Spikelet development of rice can be divided into eight stages (Sp1–Sp8) based on the initial order of floral organ primordia, and the two stigma primordia were formed at stage Sp8 (Ikeda *et al.*, 2004). In WT, the two stigma primordia are formed early in ovary development at Sp8e (early Sp8) which elongate as ovary development at Sp8l (late Sp8) stage (Figure 3g). In *ospid‐4*, however, no stigma primordium was observed (Figure 3h); eventually, a stigma‐free pistil was formed in this extreme case. Although the mutant pistils form abnormal stigmas, they seem to have normal ovules (103/104, Figure S1b). 4.1% (*n* = 737, Table S6) of *ospid‐4* pistils with large ovary were observed (Figure 3i) which often contain two ovules (Figure 3j). Two ovules contain normal embryo sac as the WT (Figure 3j). From the statistical data in Table S6, the double‐ovule phenotype was mainly observed in the pistil without stigma (20/26). These data suggest that the mutation only affects style/stigma formation but does not affect ovule development.

### Cloning of candidate gene by an improved MutMap method

**Figure 4:**
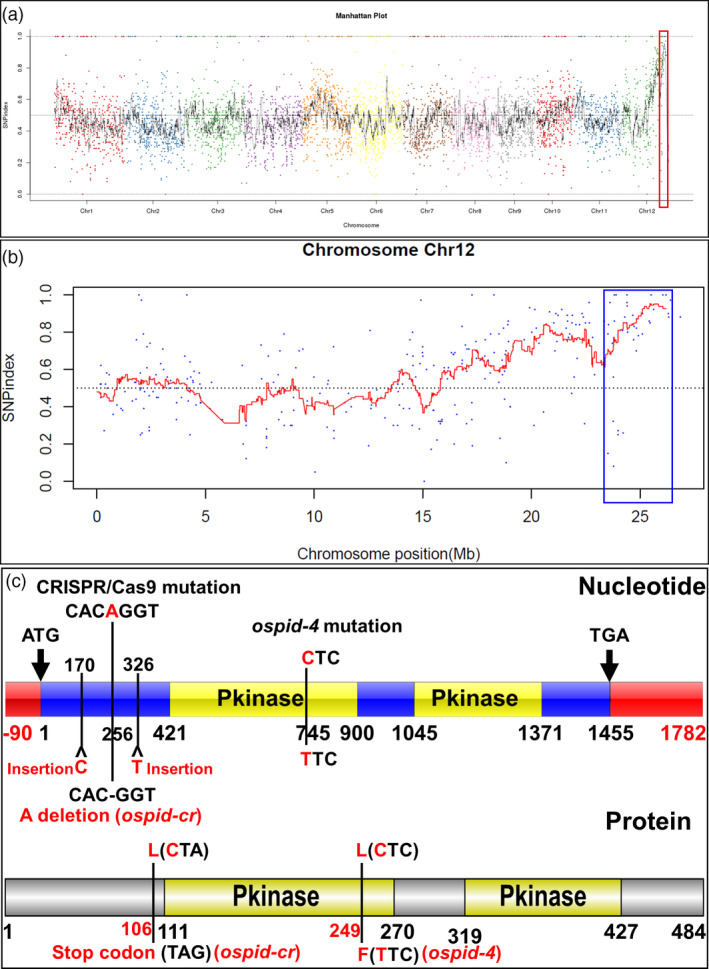
SNP‐index plot of the bulked DNA from mutant progeny and diagram of DNA and amino acid of *OsPID*. (a,b) SNP‐index plot of whole genome (a) and chromosome 12 (b) of the bulked DNA from mutant progeny. *X*‐axis, physical position; *Y*‐axis, SNP index. (c) Variation of DNA (top) and amino acid (bottom) sequence of *OsPID*. *ospid‐4* contained a C745T substitution in the CDS region of *OsPID*, resulting in a leucine to a phenylalanine (249^Leu>Phe^), *ospid‐cr* harboured a deletion of base A256 in the CDS region, which leads to a premature stop codon (106^CTA>TAG^), the other two mutations were base C170 and T326 insertions in the CDS region, resulting in a frameshift mutation in the 58th and the 110th amino acid, respectively. Red frame represents 5′ or 3′ UTR (Untranslated Regions), blue frame represents coding region, and yellow frame shows DNA sequence of Pkinase regions. Grey and yellow frames show amino acid sequence and Pkinase domains, respectively.

This mutant was derived from the EMS mutant library, so it is a good choice to identify the causal gene responsible for interpreting the mutant phenotype using the improved MutMap (Abe *et al.*, 2012). The mutant as male was backcrossed with WT parental line, and 208 F_2_ plants were obtained including 164 plants without mutant phenotype (WP) and 44 mutant plants (MP). Two DNA bulks of 30 individuals from 164 WP and 44 MP, together with DNA from WT plant, were subjected to whole‐genome sequencing. All the value of single nucleotide polymorphism (SNP) index of the bulked DNA from mutant progeny was obtained and plotted on the 12 chromosomes (Figure 4a), and a region (labelled with red frame) with SNP index of 1 or approximation was initially identified and further mapped to a 23–27 Mb region (labelled with blue frame) on chromosome 12 (Figure 4a, b). A total of ten SNPs with SNP index of 1 were picked out on chromosome 12 and associated with seven *ORFs*, of which six SNPs associated with *ORF1‐5* were located in the candidate region (23–27 Mb; Table 1). Only two SNPs from *ORF2* and *ORF6* lead to nonsynonymous substitution, a SNP at nucleotide position 26,056,055 corresponded to the exon of *ORF2* resulted in an L249F conversion in the protein sequence, and another SNP at nucleotide position 17,267,695 corresponding to the second exon of *ORF6* resulted in an amino acid variation in R620P. Genetically, the SNP with a WP‐SNP index of 0.35 from *ORF2* is more likely the causative SNP. The gene ID of *ORF2* is *LOC_Os12g42020*, which is predicted to encode a rice homologue of the *Arabidopsis PINOID* (*PID*; Morita and Kyozuka, 2007), here designated as *OsPID*. Sequence analysis showed that the mutant only contained a C745T substitution resulted in a leucine to a phenylalanine (249^Leu>Phe^) in the first kinase domain compared with WT (Figure 4c, Figure S2a). 164 WP contained homozygous C or heterozygous C/T, whereas all 44 MP harboured homozygous T, suggesting that the SNP (C745T) is linked to the phenotype of abnormal floral organs in the mutant. This shows that the SNP in *ORF2* is most likely the causative SNP.

**Table 1:** Candidate *ORFs* of the causative gene

Position (Chr.12)	WT base	Mutant base	WP‐SNP index	MP‐SNP index	Variation region	Candidate *ORFs*	Mutation types
26,201,284	T	A	0.48	1	Intronic	*ORF1*	S
26,056,055	C	T	0.35	1	Exonic	*ORF2*	NS
25,512,848	C	T	0.35	1	Intronic	*ORF3*	S
24,400,012	G	A	1	1	Intronic	*ORF4*	S
23,899,324	C	T	0.1	1	Intergenic		S
23,818,910	C	T	1	1	Downstream	*ORF5*	S
18,279,215	A	C	1	1	Intergenic		S
17,267,695	C	G	1	1	Exonic	*ORF6*	NS
4,148,836	G	T	1	1	Intergenic		S
1,935,199	C	A	1	1	Intronic	*ORF7*	S

WP‐SNP index refers to SNP index of DNA bulks from plants without mutant phenotype; MP‐SNP index refers to SNP index of DNA bulks from mutant plants. NS, nonsynonymous; S, synonymous.

### Mutation of *OsPID* is responsible for abnormal floral organ phenotype

**Figure 5:**
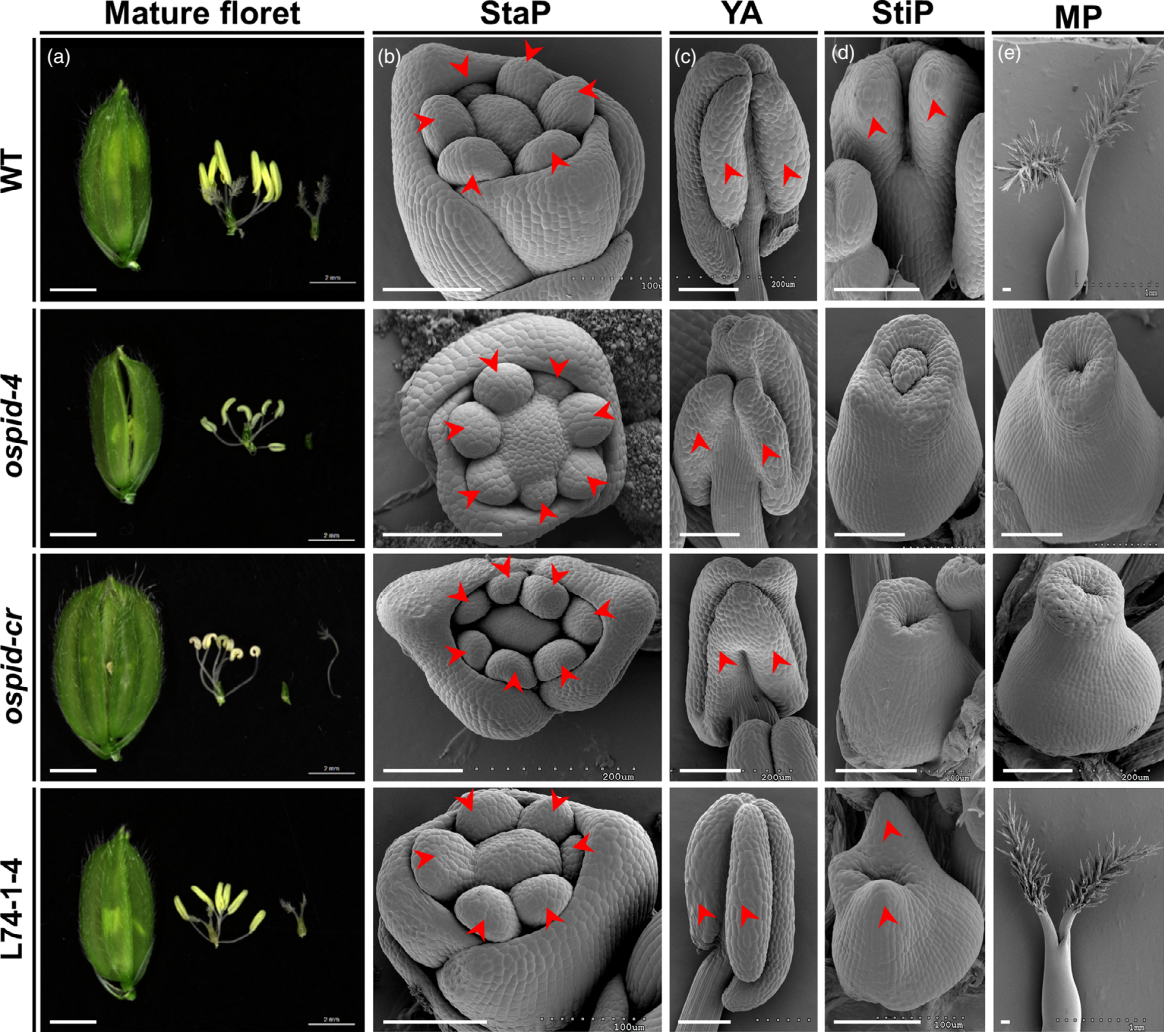
Phenotypic comparison of floral organs of WT, *ospid‐4*, *ospid‐cr* and L74‐1‐4. (a) Comparison of hull, pistil and stamen in mature florets of WT, *ospid‐4*, *ospid‐cr* and L74‐1‐4. Bars, 2 mm. (b–e) SEM analysis of stamen primordium (StaP) (b), young anthers (YA) (c), stigma primordium (StiP) (d) and mature pistil (MP) (e) in WT, *ospid‐4*, *ospid‐cr* and L74‐1‐4 florets. Red arrows in (b–d) indicate stamen primordium, pollen sac and stigma primordium, respectively. Bars, 100 μm.

To verify that *OsPID* is the target gene that causes abnormal floral organ, CRISPR/Cas9‐mediated gene editing and genetic complementation approaches were employed. Firstly, three homozygous lines harbouring three types of mutations by CRISPR/Cas9‐mediated gene editing technology were obtained. The first type mutation was a deletion of base A256 that led to a premature stop codon (106^CTA>TAG^) at 316b downstream of the deletion (Figure 4c, Figure S2a), and this mutation was designated as *ospid‐cr*. The other two mutations were single base insertions resulting in a frameshift, inserting a C and T after the 170th and 326th bases of the coding region (Figure 4c), resulting in a frameshift mutation in the 58th and the 110th amino acid, respectively. All three types of mutations caused deletion of the two Pkinase regions (Figure 4c) of *OsPID*. The phenotypic analysis on *ospid‐cr* plant, which was stable and CRISPR/Cas9‐free, showed that the mutation phenocopied *ospid‐4* phenotype (Figure 5; Tables S1, S4, S5, S7). Secondly, DNA fragment consisting of the entire CDS, the upstream 5010 bp and the downstream 2002 bp DNA sequence of *OsPID* (Figure S2b) were transformed into the *ospid‐4* mutant, and six independent and stable transgenic lines were obtained. Phenotypic analysis on transgenic lines L74‐1‐4, L74‐2‐4 and L74‐3‐1 showed that the DNA fragment completely rescued the mutant phenotype (Figure 5, Figure S2c; Tables S8 and S9). These results indicated that *OsPID* was the gene responsible for the phenotypic variation in the *ospid‐4* mutant.

### *OsPID* gene is expressed in floral meristem and organ primordia

**Figure 6:**
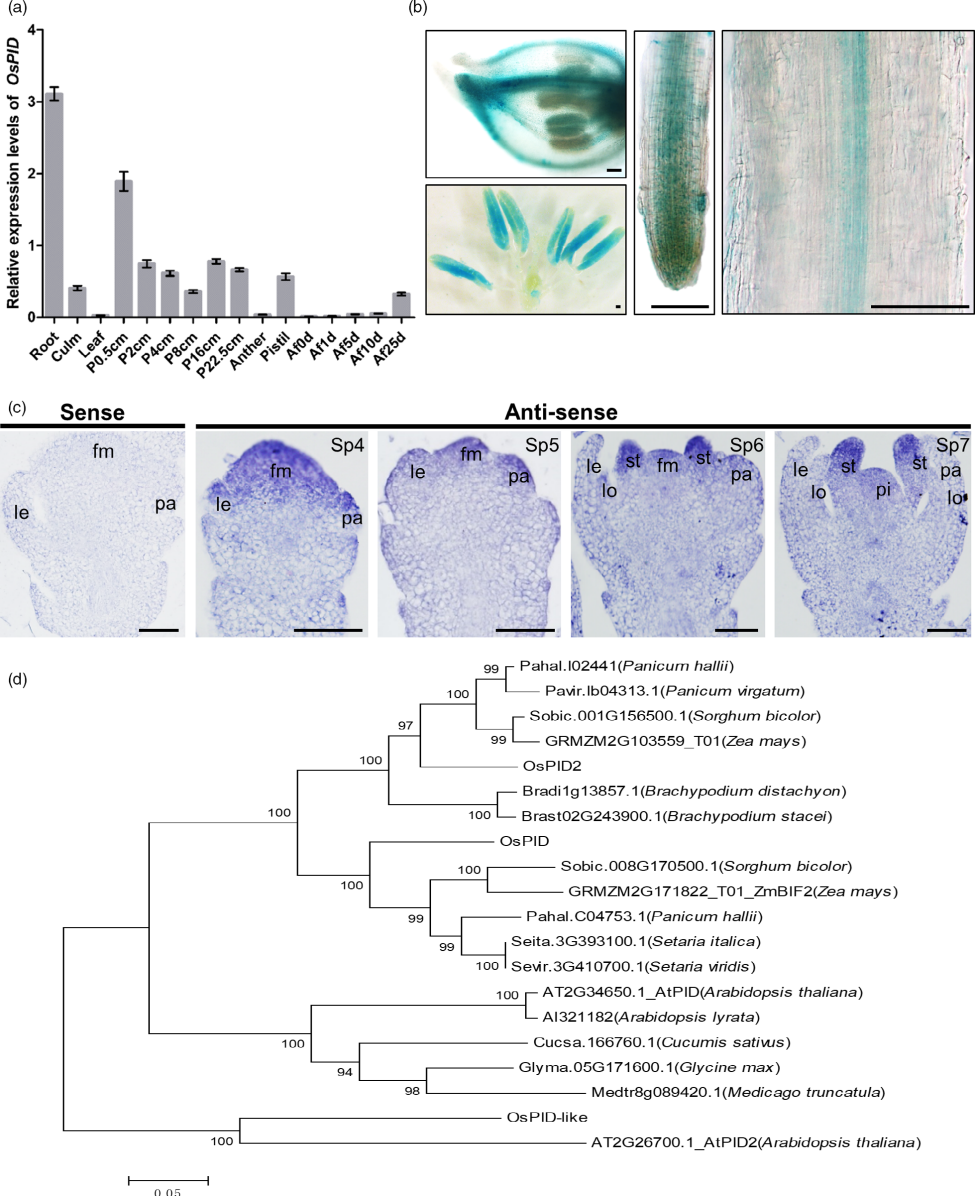
The expression pattern of *OsPID* and phylogenetic analysis of OsPID homologs. (a) qRT‐PCR analysis of *OsPID* expression pattern. P 0.5 cm, P 2 cm, P 4 cm, P 8 cm, P 16 cm and P 22.5 cm represent panicle of 0.5, 2, 4, 8, 16 and 22.5 cm, respectively; Af 1 days, Af 5 days, Af 10 days and Af 25 days represent panicle of 1 day, 5 days, 10 days and 25 days after fertilization, respectively. Values are means ± SD of three biological repeats. (b) Analysis of *OsPID* expression pattern in *OsPID::OsPID‐GUS* transgenic plants. GUS staining in floret (upper left), anthers (lower left) and roots (middle and right). Bars, 100 μm. (c) *In situ* hybridization analysis of *OsPID* expression pattern. Negative control from hybridization with *OsPID* sense probe (left‐most). RNA *in situ* hybridization with *OsPID* antisense probes in ZH11 florets at Sp4, Sp5, Sp6 and Sp7. fm, floral meristem; le, lemma; pa, palea; lo, lodicule; st, stamen; pi, pistil. Bars, 50 μm. (d) Phylogenetic analysis of OsPID homologs from different species. Phylogenetic tree was constructed using MEGA5.0 by neighbour‐joining (NJ) method. All amino acid sequences were retrieved from Phytozome12 (https://phytozome.jgi.doe.gov/pz/portal.html): *Pahal.I02441* (*Panicum hallii*); *Pavir.Ib04313.1* (*Panicum virgatum*); *Sobic.001G156500.1* (*Sorghum bicolor*); *GRMZM2G103559_T01* (*Zea mays*); *Bradi1g13857.1* (*Brachypodium distachyon*); *Brast02G243900.1* (*Brachypodium stacei*); *Sobic.008G170500.1* (*S. bicolor*); *GRMZM2G171822_T01* (*Zea mays*); *Pahal.C04753.1* (*P. hallii*); *Seita.3G393100.1* (*Setaria italica*); *Sevir.3G410700.1* (*Setaria viridis*); *AT2G34650.1* (*Arabidopsis thaliana*); *Al321182* (*Arabidopsis lyrata*); *Cucsa.166760.1* (*Cucumis sativus*); *Glyma.05G171600.1* (*Glycine max*); *Medtr8g089420.1* (*Medicago truncatula*); *AT2G26700.1* (*Arabidopsis thaliana*).

To explore the expression pattern of *OsPID* gene, RT‐PCR and transgenic approach and RNA *in situ* hybridization were employed. The results showed that *OsPID* gene is expressed at the highest level in roots and hardly any in leaf. During flower development, *OsPID* is most highly expressed in panicles at 0.5 cm length and throughout the panicle development as well as in pistil. Its expression is very low in inflorescence after flowering (Figure 6a). Transgenic study with GUS reporter also showed that *OsPID* is expressed in hull, anther and root (Figure 6b). RNA *in situ* hybridization further showed that *OsPID* is expressed in floral meristem, lemma and palea primordia at Sp4‐5 stages, later, and its expression remains in stamen and pistil primordia but disappears in young lemma and palea (Figure 6c). These indicated that *OsPID* expression coincides with floral organ primordium development during flower development in rice.

Sequence alignment analysis revealed that *OsPID* has two homologues in rice, namely *OsPID*‐like and *OsPID*2. We selected the top 20 proteins with high homology for phylogenetic analysis and found that the homology between OsPID and PID in sorghum and maize was the closest (Figure 6d). Sequence alignment of the 20 protein sequences revealed that the two Pkinase regions of PID are highly conserved in each species. The region of red line labelled in Figure S3 was the two Pkinase domains of PID, and the amino acid mutated (249^Leu^) highlighted in blue frame located in the conserved Pkinase domain (Figure S3). To verify whether OsPID was functionally conserved in *Arabidopsi*s, we transferred *AtPID::OsPID* to two mutant lines *atpid‐1* and *atpid‐3*, which had abnormal floral organs. The transgenic plant L2‐6 could restore the phenotype of the dwarf, excessive petals and malformed siliques of the *pid‐1* mutant (Figure S4a–c). Similarly, the phenotype of pin‐like inflorescence, excessive petals and malformed siliques of the *pid‐3* mutant was also rescued in the transgenic plant L4‐5 (Figure S4d–f). These results demonstrated that the function of PINOID in rice and *Arabidopsis* is conserved and further confirmed that *OsPID* is responsible for phenotypic variation.

### Auxin distribution is impaired during floral organ development in *ospid‐4*

**Figure 7:**
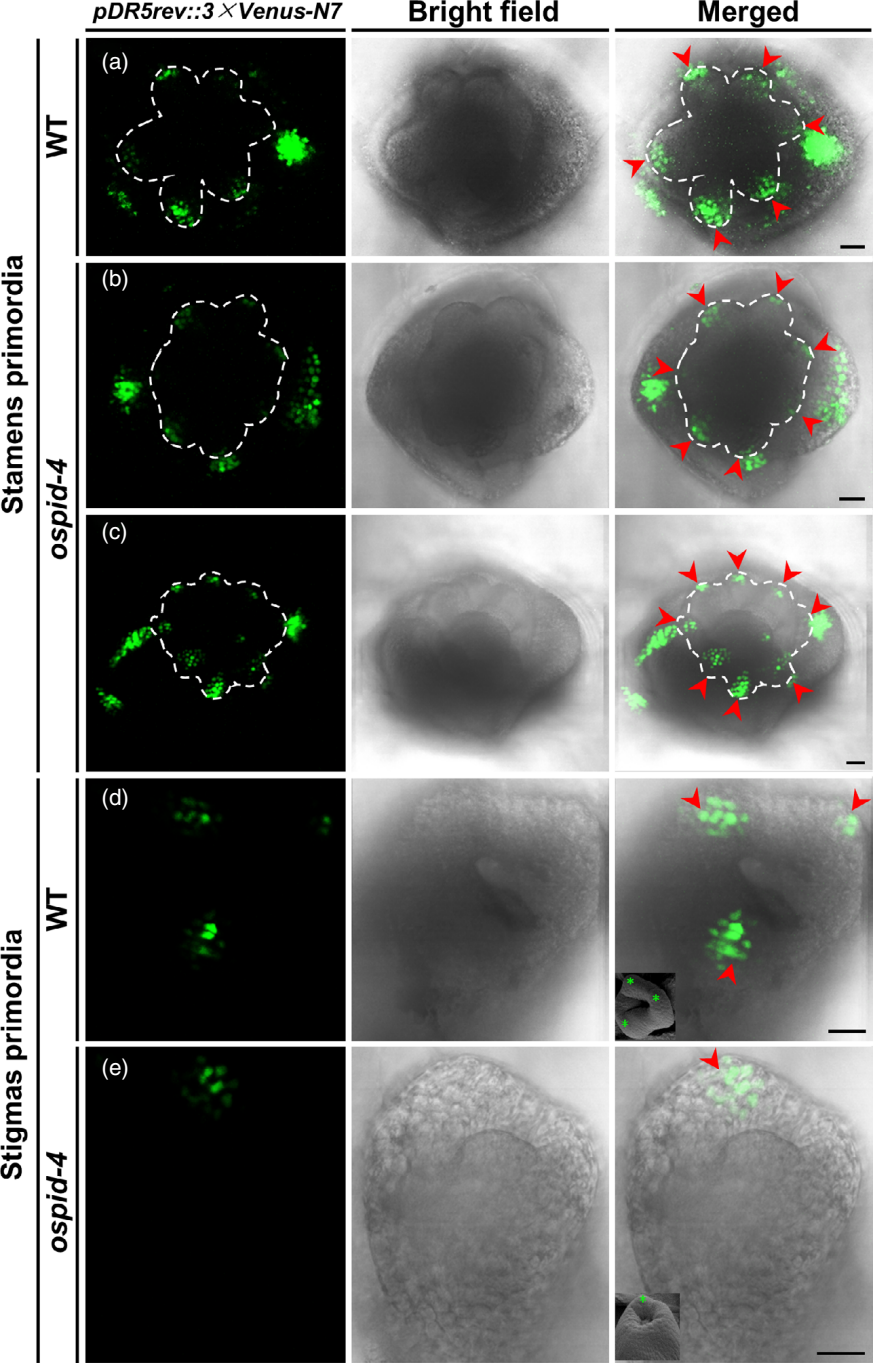
Auxin distribution in stamen and stigma primordia of WT and *ospid‐4*. (a) Confocal images show auxin distribution in six stamen primordia of WT. (b,c) Confocal images show auxin distribution in seven (b) or eight (c) stamen primordia of *ospid‐4*. (d) Confocal images show auxin distribution in three stigma primordia of WT. (e) Confocal images show auxin distribution in a stigma primordium of *ospid‐4*. *pDR5::VENUS* signal (left column), bright field image (middle column) and *pDR5::VENUS* signal was merged with bright field image (right column). The stamen primordium is labelled with the white dash line and red arrow in (a–c), and the stigma primordium is labelled with red arrow in (d,e), SEM images of stigma primordia labelled with green stars in the right column of (d,e). Bars, 20 μm.

Previous researches showed that PINOID regulates the polar distribution of auxin by controlling subcellular localization of PIN auxin efflux carriers in *Arabidopsis* (Friml *et al.*, 2004; Lee and Cho, 2006). The primordium development of *ospid‐4* stigmas and stamens is abnormal, given that the function of PINOID in rice and *Arabidopsis* is conserved; therefore, defects in stamens and pistils of the *ospid‐4* mutant are likely due to the disruption of the polar distribution of auxin. To verify this hypothesis, we used the *DR5* reporter system (*pDR5rev::3 × Venus‐N7*) to observe the distribution of auxin in pistil and stamen primordia. At the stage of stamen primordium formation, six *VENUS* signal‐concentrated regions were observed in WT (Figure 7a), corresponding to six stamen primordia, while seven (Figure 7b) or eight (Figure 7c) *VENUS* signal‐concentrated regions were found in the *ospid‐4* mutant, corresponding to seven or eight stamen primordia, which indicates that the polar distribution of auxin in the stamens of the *ospid‐4* mutant is disturbed, thereby forming more stamen primordia than the WT. Similarly, during the formation of the WT stigma primordium, three *VENUS* signal‐concentrated regions were detected (Figure 7d), corresponding to three stigma primordia (the latter one stigma will degenerate), and no or only one *VENUS* signal‐concentrated regions was found (Figure 7e) in *ospid‐4*, corresponding to the pistil without style or single style/stigma, which indicates that the polar distribution of auxin in *ospid‐4* pistil is also disordered, resulting in the formation of pistils with little or no stigma primordium. Together, these results indicated that abnormal stamens and pistils of the *ospid‐4* mutant are likely caused by the disruption of the polar distribution of auxin.

### OsPID is localized to the nucleus, cytoplasm and plasma membrane

**Figure 8:**
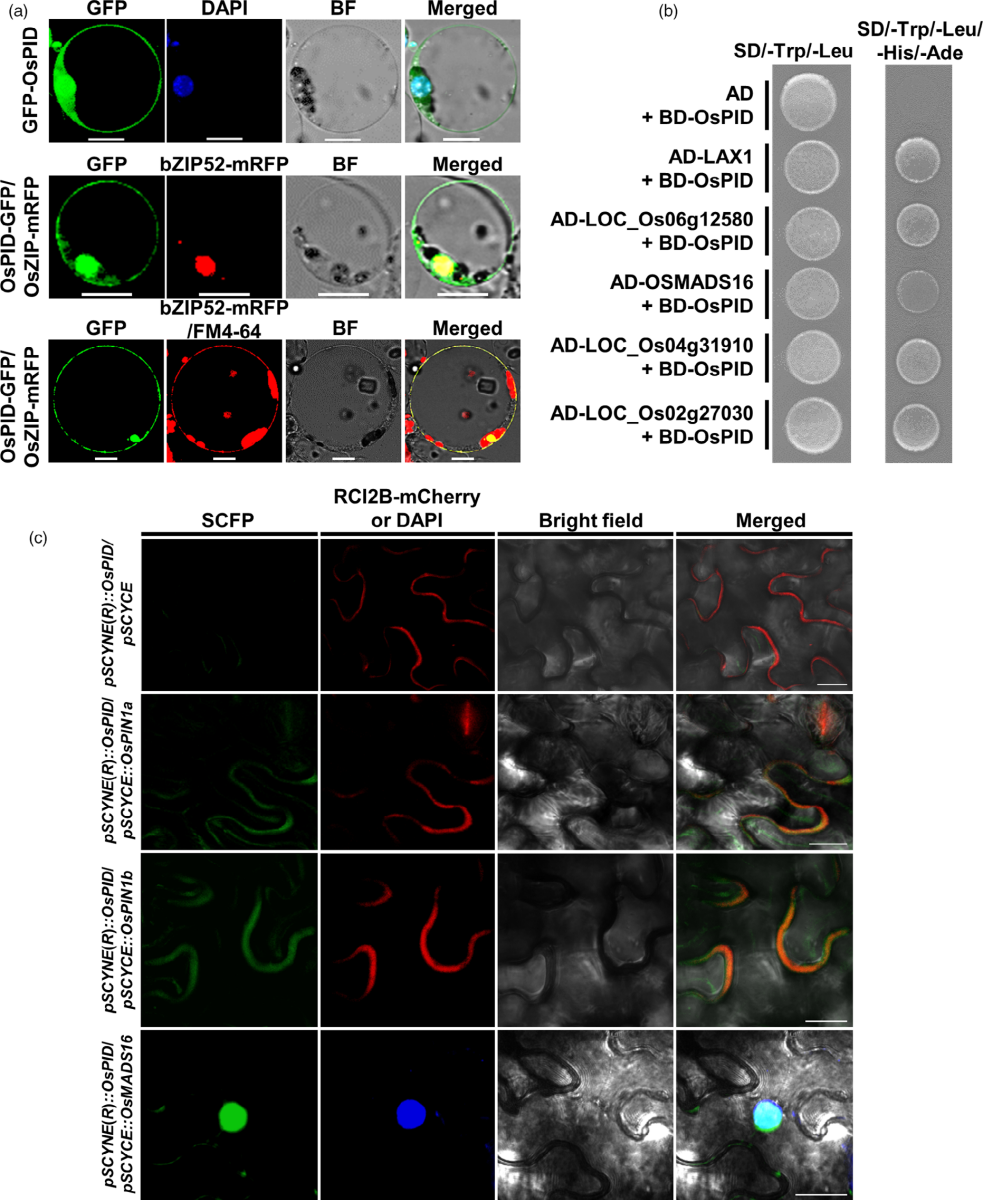
Subcellular localization and interaction analysis of OsPID. (a) Subcellular localization of OsPID in rice protoplasts. GFP‐OsPID is co‐localized with DAPI (a dye of nuclei) in the nucleus (top row), OsPID‐GFP is co‐localized with bZIP52‐mRFP (a nuclear marker) in the nucleus (middle row) and FM4‐64 (a dye of plasma membrane) on the plasma membrane (bottom row). Bars, 10 μm. (b) Verification of interaction between OsPID and candidate proteins with Y2H. OsPID can interact with LAX1, LOC_Os06g12580, OsMADS16, LOC_Os04g31910 and LOC_Os02g27030 in yeast, respectively. SD/‐Trp/‐Leu, Synthetic dropout medium without Trp and Leu; SD/‐Trp/‐Leu/‐Ade/‐His, Synthetic dropout medium without Trp, Leu, Ade and His. AD, *pGADT7*; BD, *pGBKT7*. Empty vector *pGADT7* plus *pGBKT7*‐OsPID was used as the control. (c) BiFC assay of the interactions between OsPID and OsPIN1a, OsPIN1b and OsMADS16 in tobacco leaves. Confocal images show OsPID interacts with OsPIN1a (the second row) and OsPIN1b (the third row) on the plasma membrane. Confocal images show OsPID interacts with OsMADS16 (bottom row) in the nucleus. SCFP signal (left column), RCI2B‐mCherry (an intrinsic plasma membrane protein) or DAPI signal (the second column from left to right), bright field image (the third column from left to right), SCFP signal was merged with RCI2B‐mCherry signal and bright field image or with DAPI signal and bright field image (right column). Bars, 20 μm.

To verify the subcellular localization of OsPID, GFP fusion with OsPID was made and introduced into rice protoplasts together with either the nuclear marker OsbZIP52‐mRFP or the plasma membrane marker FM‐4‐64. The results showed that OsPID is localized to cytoplasm (Figure 8a). It is also localized in plasma membrane and nucleus manifested as its co‐localization with FM‐4‐64 and OsbZIP52‐mRFP, respectively (Figure 8a). To further confirm the subcellular localization of the OsPID, we also observed roots expressing *pUBI::GFP‐OsPID* and *pUBI::OsPID‐GFP*, and found that both GFP‐OsPID and OsPID‐GFP are localized to nucleus, cytoplasm and plasma membrane (Figure S5a,b). Together, these results indicated that OsPID is localized to the nucleus, cytoplasm and cell membrane.

### OsPID interacts with OsMADS16, OsPIN1a and OsPIN1b

**Figure 9:**
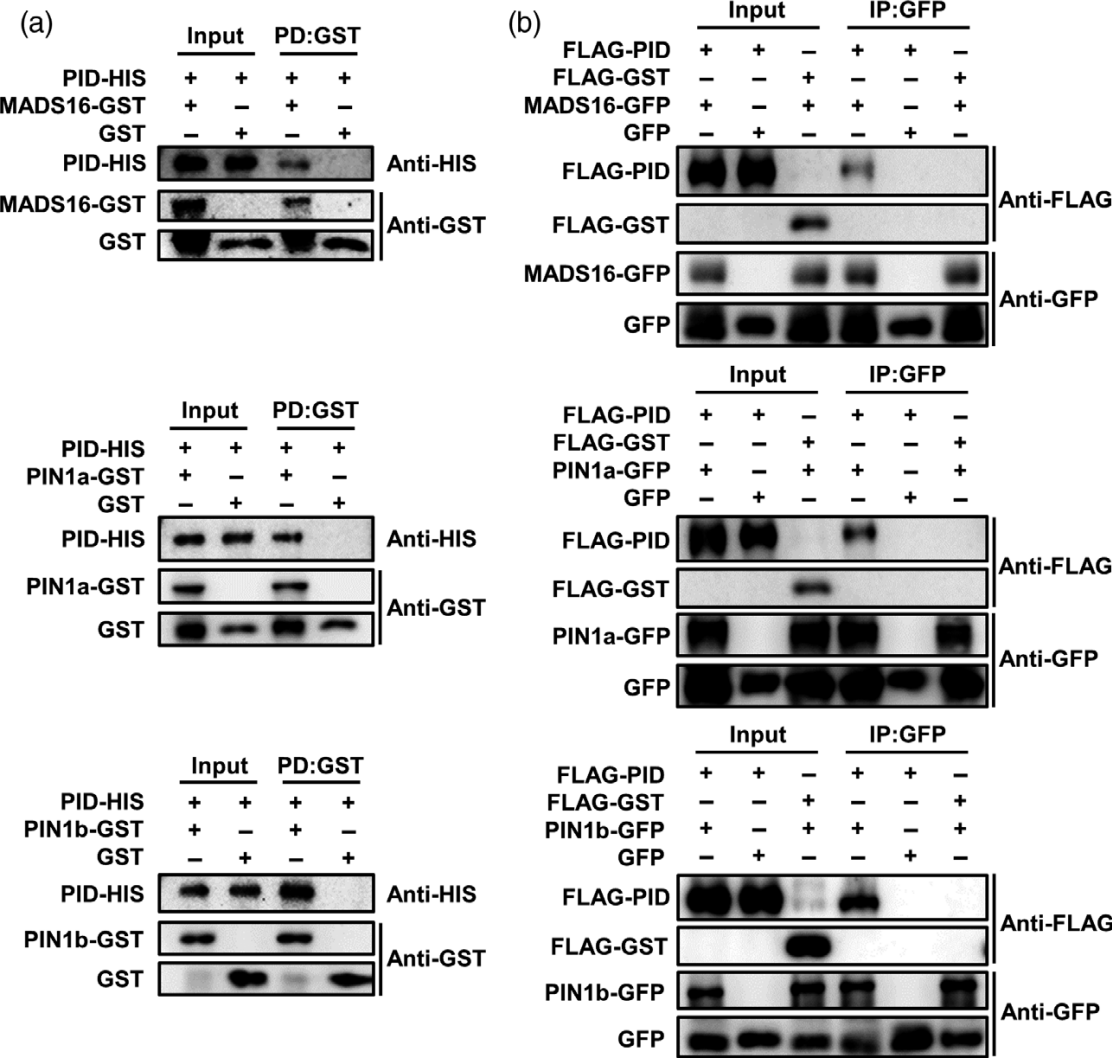
OsPID interacts with OsMADS16, OsPIN1a and OsPIN1b *in vitro* and *in vivo*. (a) *In vitro* pull‐down assay verified the interactions between OsPID and OsMADS16 (top row), OsPIN1a (middle row) and OsPIN1b (bottom row). Left panel (Input), before precipitation; Right panel (PD), Pull‐down. (b) *In vivo* Co‐IP assay validates the interactions between OsPID and OsMADS16 (top row), OsPIN1a (middle row) and OsPIN1b (bottom row). GFP and FLAG‐GST were used as negative controls. Left panel (Input), before precipitation; Right panel (IP), co‐immunoprecipitation.

To further explore the molecular mechanism of OsPID in regulating the development of pistil and stamen in rice, an Y2H screen for proteins that interact with OsPID was performed. Our data showed that OsPID can interact with five proteins, LAX1, LOC_Os06g12580, OsMADS16, LOC_Os04g31910 and LOC_Os02g27030 (Figure 8b). To further confirm the interaction of OsPID‐OsMADS16, a bimolecular fluorescence complementary (BiFC) assay was performed. Indeed, OsPID interacts with OsMADS16 (Figure 8c) in the nucleus of tobacco (*Nicotiana tabacum* L.) epidermal cells. Furthermore, we confirmed the interaction between OsPID and OsMADS16 by *in vitro* pull‐down assay (Figure 9a) and *in vivo* co‐immunoprecipitation (Co‐IP) assay (Figure 9b). This suggests that OsPID may regulate floral organ development of rice by interacting with the transcription factor OsMADS16.

In *Arabidopsis*, PINOID controls the subcellular localization of PIN by phosphorylating auxin efflux carrier PIN and then regulates the polar distribution of auxin (Friml *et al.*, 2004; Lee and Cho, 2006; Michniewicz *et al.*, 2007). In rice, *OsPIN1a*, *OsPIN1b*, *OsPIN1c* and *OsPIN1d* were identified as the homologous gene of *PIN1* in *Arabidopsis* (Wang *et al.*, 2009). To investigate whether OsPID also interacts with the OsPIN1 to regulate the transport of auxin, we performed a BiFC assay in tobacco leaves, and our data showed OsPID interacts with OsPIN1a and OsPIN1b (Figure 8c) on the cell membrane. Furthermore, the interactions between OsPID and OsPIN1a, OsPIN1b were confirmed by *in vitro* pull‐down assay (Figure 9a) and *in vivo* Co‐IP assay (Figure 9b). These results indicate that OsPID may regulate polar auxin transport to control floral organ development by interacting with OsPIN1a and OsPIN1b.

## Discussion

### *OsPID* controls floral organ development in rice

In *Arabidopsis*, *PID* is a classic gene involved in the origination of flower primordium. Many *pinoid* mutants, including strong, intermediate and weak allelic mutants, have been identified. Among these mutants, strong mutants of *PID* form pin‐like inflorescences, and other pleiotropic phenotypes on floral organ development, cotyledons and leaves have also been found (Bennett *et al.*, 1995). So far, 15 *pid* mutants (*pid‐1* to *pid‐15*) have been identified and characterized in *Arabidopsis* (Lin *et al.*, 2017). In this study, we identify an allelic mutant (*ospid‐4*) of *PID* in rice, which has a pleiotropic phenotype on floral organ development and phenotypically differs from several allelic mutants of *PID* (He *et al.*, 2019; Xu *et al.*, 2019), such as the abnormal hulls and defective pollen grains. The phenotype of more than six stamens and pistil with fewer or no style/stigma in *ospid‐4* mutant is different from other mutants with an abnormal number of floral organs, such as the *fon1*, *fon2* and *fon4* mutants, which have an increased number of floral organs (Chu *et al.*, 2006; Suzaki *et al.*, 2004; Suzaki *et al.*, 2006). Different from the normal pollen grains of *ospid‐1* mutant (Xu *et al.*, 2019), only 62.1% pollen grains of *ospid‐4* can be positively stained with I_2_‐KI. The seed setting rate of the *ospid‐4* mutant is 2.2%, rather than complete abortion, which is conducive to the preservation of the mutant. Furthermore, about 4.1% of *ospid‐4* pistil have double ovules and normal embryo sacs (103/104, Figure S1b). However, in the *OsIG1*‐RNAi plants, about 40% of ovaries have double ovules with abnormal embryo sacs (Zhang *et al.*, 2015), and double‐seed in a floret is not found. In *fon3* mutants, the florets have an average of four ovules, and two or three small seeds in a single floret are found frequently (Jiang *et al.*, 2005). The phenotype of double‐seed in one floret is not found because *ospid‐4* pistil with double ovules often lacks stigmas. If the percentage of double‐ovule phenotype is large in florets, in which normal seeds are formed, this will increase the yield potential.

In the recent two studies, all the pistils of *pid* mutants are absent of stigma, and it means that these *pid* mutants may be strong allelic mutants (He *et al.*, 2019; Xu *et al.*, 2019). However, 76% of *ospid‐4* pistils are stigma‐free, 16.7% show double stigmas with less stigma hair, and 6.8% show single stigma with less stigma hair, suggesting *ospid‐4* mutant is not a strong allelic mutant. Our results indicated that *ospid‐cr* plant showed a similar or even more severe phenotype to the *ospid‐4* mutant, suggesting *ospid‐cr* may be a strong allelic mutant. The phenotype of *ospid‐cr* plant is similar to that of two CRISPR/Cas9 editing lines *ospid‐c1* and *ospid‐c2* (He *et al.*, 2019). These mutants*, ospid‐1* (a 34‐bp deletion in the coding region), *ospid‐2* (a T631 deletion), *ospid‐3* (a base A insertion between nucleotides 1265 and 1266), *ospid‐1* (E164K), *ospid‐c1* (a 47‐bp deletion in the coding region) and *ospid‐c2* (a 4‐bp deletion in 267b of CDS region), all show no stigma completely (He *et al.*, 2019; Xu *et al.*, 2019). Nevertheless, a part of *ospid‐2* (S320L), *ospid‐4* (L249F) and *ospid‐cr* (a base A256 deletion) pistils have one or two stigmas (He *et al.*, 2019), it is unclear why there are such big phenotypic variations. In maize, *bif2* mutant also developed pin‐like inflorescences with fewer branches and floral organs (McSteen and Hake, 2001; McSteen *et al.*, 2007). Although a number of *pid* mutants have been identified and characterized, the underlying mechanism is still unclear.

The application of male sterility in rice hybrid breeding has been widely studied (Chang *et al.*, 2016; Kim and Zhang, 2018; Li *et al.*, 2007), while few studies reported the role of female sterility in rice cross‐breeding (Qu *et al.*, 2012). The phenotype of pistil without stigma or style in *ospid‐4* mutant will effectively promote rice hybrid breeding by planting of two sterile parents to produce hybrids (Qu *et al.*, 2012; He *et al.*, 2019).

### Possible mechanism of OsPID in regulating rice floral organ development

The phenotype of the *pid* allelic mutations as discussed above clearly indicates that PID plays a pleiotropic role in flower and floral organ development. It was established that PID acts as a kinase to phosphorylate the auxin efflux carrier PIN, thereby positively modulating polar auxin transport in *Arabidopsis* (Friml *et al.*, 2004; Michniewicz *et al.*, 2007). Since *AtPID::OsPID* construct can rescue the phenotype of two mutant lines *atpid‐1* and *atpid‐3*, *PID* is functionally conserved between rice and *Arabidopsis*. Furthermore, OsPID interacts with OsPIN1a and OsPIN1b as in maize and *Arabidopsis* (Skirpan *et al.*, 2009; Zourelidou *et al.*, 2014). Therefore, PID most likely regulates rice floral organ development through the auxin pathway. Indeed, polar distribution of auxin was disrupted in *ospid‐4* mutant as revealed by *DR5* reporter system. In *ospid‐4* flower primordia, there are more centres with auxin maximum, which coincides with the number of stamen and stigma observed. This suggests that disruption of auxin polar transport early in flower development is the main cause of the observed abnormal floral organs. Taken together, OsPID most likely regulates polar auxin transport to control floral organ development by interacting with OsPIN1a and OsPIN1b in rice.

In addition to the auxin pathway, OsPID may also regulate rice floral organ development by interacting with transcription factors. Firstly, different from AtPID in *Arabidopsis* (Lee and Cho, 2006; Michniewicz *et al.*, 2007; Zegzouti *et al.*, 2006), OsPID is localized to the nucleus, cytoplasm and cell membrane in rice protoplasts and transgenic plant roots. This is similar to the localization of ZmPID in maize (Skirpan *et al.*, 2008). Its nuclear localization implies that OsPID may regulate transcription. Indeed, OsPID interacts physically with OsMADS16, which acts as an antagonist to DL and controls the identity of stamen and carpel in rice (Nagasawa *et al.*, 2003). The stamens of the *osmads16* mutant develop ectopically into carpels (Moon *et al.*, 1999), and a stigma growing ectopically on the filament is observed in *ospid‐cr* mutant (Figure 5a). Furthermore, OsMADS16 can interact with OsMADS3 and OsMADS58 to determine the identity of rice floral organs (Yun *et al.*, 2013).

**Figure 10:**
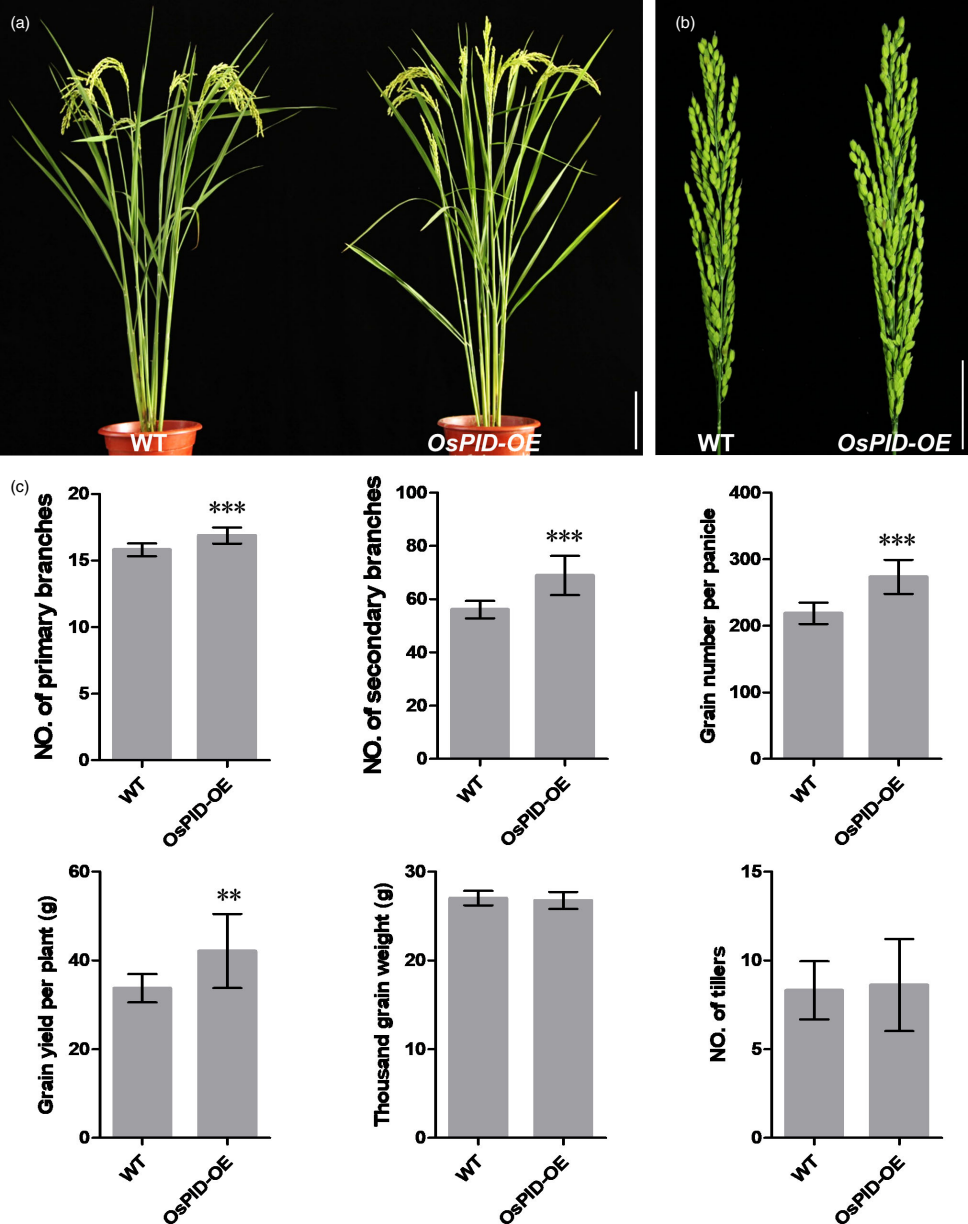
Phenotype of WT and *OsPID‐OE* (an *OsPID* overexpression line expressing *pUBI::OsPID* vector) (a) Morphological comparison of WT and *OsPID‐OE*. Bar, 10 cm. (b) Panicle comparison of WT and *OsPID‐OE*. Bar, 5 cm. (c) Comparison of primary branch number, secondary branch number, grain number per panicle, grain yield per plant, thousand grain weight and tillers number per plant between WT and *OsPID‐OE*. Values are means ± SD (*n* = 10). ** and *** represent significant differences at *P*‐value 0.01 and 0.001, respectively.

LAX1 is an important regulator for initiation of axillary meristems in rice, which encodes a transcription factor containing a basic helix‐loop‐helix (bHLH) domain and is transiently expressed in axillary meristems (Komatsu *et al.*, 2001). Recent work has shown that LAX2/Gnp4 regulates rice grain length by interfering with the interaction of OsIAA3‐OsARF25 (Zhang *et al.*, 2018). Therefore, LAX1 may also be involved in the auxin regulation pathway. Y2H assay showed OsPID can interact with LAX1 in yeast. In maize, the homologous protein of PID, ZmBIF2, co‐localizes in the nucleus with ZmBA1 (homologous protein of OsLAX1), so it is deduced that BIF2 also plays a role in the nucleus, in addition to regulating the transport of auxin around the cell, and regulates the initiation of axillary meristems by interacting with BA1 (Skirpan *et al.*, 2008). LAX1 interacts with LAX2 to regulate the development of leaf axillary meristems (Tabuchi *et al.*, 2011; Zhang et al., 2011c), suggesting that auxin may be involved in the regulation of differentiation of axillary meristems by LAX1 and LAX2. *ospid osnpy2* double mutants form a pin‐like inflorescences, fewer tillers and no flowers (He *et al.*, 2019), suggesting that OsPID may be involved in the regulation of branches, tillers and flower initiation. We also found an *ospid‐cr2* mutant with fewer branches compared with WT (Figure S5c,d). Moreover, overexpression of *OsPID* increased primary branch number, secondary branch number, grain number per panicle and grain yield per plant, but did not change thousand grain weight and tiller number per plant (Figure 10a–c); it suggests that *OsPID* has potential applications in high‐yield breeding of rice.

**Figure 11:**
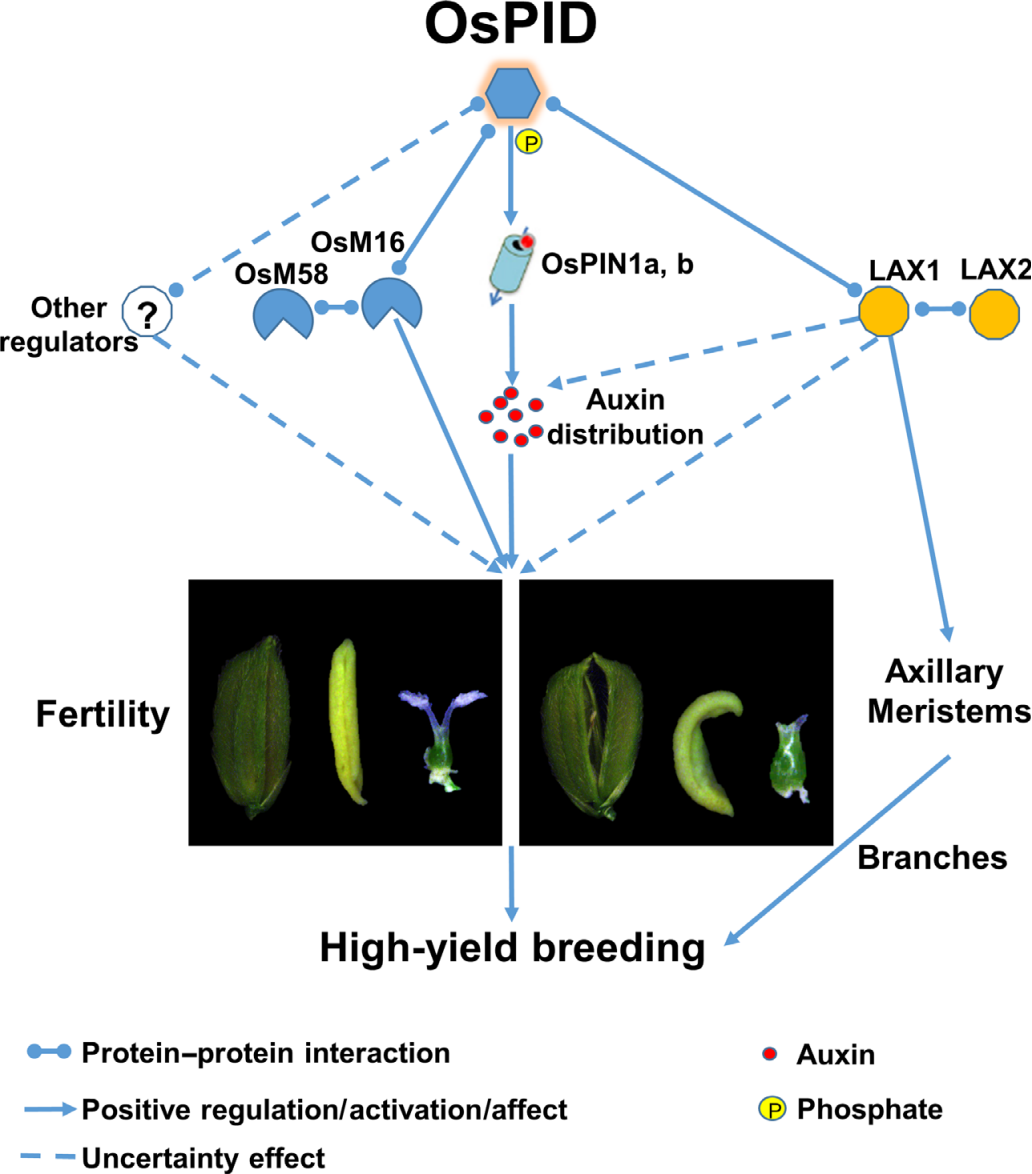
A proposed model of OsPID in regulating rice floral organ development. Firstly, OsPID, a central modulator from auxin to floral organs in rice, most probably regulates polar auxin transport to control floral organ development by phosphorylating OsPIN1a and OsPIN1b. In addition, our data revealed a novel pathway that OsPID likely regulates floral organ development of rice by interacting with the transcription factor OsMADS16. On the other hand, OsPID possibly interacts with LAX1 to regulate the development of floral organs through the auxin regulatory pathway, or OsPID may be involved in controlling rice branches and tillers to affect rice yield by interacting with LAX1, suggesting that OsPID has a potential in high‐yield breeding of rice. In addition, the existence of other regulators, such as LOC_Os02g27030 and LOC_Os06g12580, cannot be excluded.

Taking together, we propose a model that OsPID regulates floral organ development possibly through modulating polar auxin transport and interacting with OsMADS16 and/or LAX1 in rice (Figure 11). Our findings provide new insights into the molecular functions of *OsPID* in floral organ development of rice, especially in stigma development, and provide useful information for potential genetic improvement of high‐yield breeding in rice.

## Materials and Methods

### Plant materials and growth conditions

The *ospid‐4* mutant was screened from an EMS mutant library in the Zhonghua 11 (ZH11) (*Oryza sativa* ssp. *Japonica* cv.) background. The WT plants used in this study are ZH11. The *ospid‐cr and ospid‐cr2* mutants were generated by Biogle Biotechnology (Changzhou, China) using CRISPR‐Cas9‐mediated editing technology (Lu *et al.*, 2017). All rice plants were grown in paddy field under natural conditions in Beijing and Hainan.

### SEM

The fresh inflorescences and florets from living plants were immediately dissected with tweezers and mounted on the holder with 3M electrically conductive double‐sided adhesive tape, and the holder was quickly transferred into liquid nitrogen. The sample was observed and photographed with HITACHI S‐3000N&Quorum PP3000T scanning electron microscope (Hitachi, Japan).

### Microscopy

To test the pollen viability, mature florets before flowering were fixed in 70% (v/v) ethanol and then anthers from the florets were dissected and stained with 1% (w/v) I_2_‐KI on a glass slide. The number of the viable pollen grains (deeply stained and round shaped) and inviable pollen grains (lightly stained, small and shrivelled) was counted under a bright field microscope (Axioskop 2 Plus; Zeiss). Three shots were selected for each slide for the statistics of I_2_‐KI staining rate of pollen grains.

For histological analysis, anthers of different developmental stages were fixed in 4% paraformaldehyde in 0.025 M sodium phosphate buffer (pH 6.8) overnight at 4 °C. The samples were washed in PBS and dehydrated by washing them through conventional ethanol series of 30 min each and embedded in HISTORESIN (LEICA, cat#702218500) according to the manufacturer’s instructions. 4‐μm sections were cut with a Leica microtome and stained with 0.1% toluidine blue, then observed and photographed with Zeiss Axioskop 2 Plus fluorescence microscope.

### Whole‐mount staining and clearing of ovules

The structure of mature embryo sacs of WT and *ospid‐4* mutants was observed using whole‐mount stain‐clearing laser scanning confocal microscopy (WCLSM; Zhao *et al.*, 2013). The mature florets before flowering were fixed in FAA solution (50% ethanol: glacial acetic acid: 37% formalin = 89 : 6 : 5 mL), then placed under vacuum until all samples sunk to the bottom of the tube at 4 °C. The samples were fixed in fresh FAA solution for at least 24 h at room temperature (15–25 °C) and kept in 70% ethanol at 4 °C until use. The ovaries were dissected from florets in 70% ethanol then passed through an ethanol series (50%, 30% and 10%; 20 min each grade) and double‐distilled water (ddH_2_O). Subsequently, the samples were sequentially stained in 2% AlK (SO_4_)_2_∙12 H_2_O for 20 min, 10 mg/L Eosin B (in 4% sucrose) for about 12 h and 2% AlK (SO_4_)_2_∙12 H_2_O for 20 min at room temperature (15–25 °C), then rinsed at least 3 times with ddH_2_O. Thereafter, the samples were dehydrated in a graded ethanol series: 10%, 30%, 50%, 70%, 90% and 100% (three changes), 20 min each grade. The ovaries were cleared in 50% methyl salicylate (in ethanol) for 2 h and then in 100% methyl salicylate for more than 10 h. Finally, the ovaries were observed using confocal laser scanning microscope (Zeiss LSM 510 Meta).

### Mapping of rice PINOID

We performed gene mapping using a modified MutMap method (Abe *et al.*, 2012). Two DNA bulks of 30 individuals from 164 WP and 44 MP, together with a DNA from ZH11 plants (WT), were extracted and sent to Novogene Biotechnology (Beijing, China) for whole‐genome resequencing. Three libraries (WP, MP and WT) were constructed and sequenced by Illumina HiSeq2500 platform, respectively. 150 bp paired‐end reads from WP‐bulk (15.9 Gb) and MP‐bulk (13.7 Gb) were generated and aligned to the WT (7.4 Gb) reference genome with the BWA software (Li and Durbin, 2009). SNP calling was performed with the Unified Genotyper function in GATK 3.3 software (McKenna *et al.*, 2010). SNPs or insertion–deletion (InDels) were annotated by ANNONAR (Wang *et al.*, 2010) based on the GFF3 files for the reference genome. The SNP index of each SNP was calculated, which is the ratio of short reads of DNA bulks harbouring SNPs different from the reference (Abe *et al.*, 2012). An average of SNP index of SNPs (represented by a line) in a given genomic interval was calculated by using a sliding window method with window size of 1Mb and step size of 1 Kb. Two SNP‐index plots (WP‐SNP‐index and MP‐SNP‐index) were generated. A region with SNP index of 1 or approximation on chromosome 12 was picked up in the MP‐SNP‐index plot. We need to find such SNPs, the MP‐SNP index of which is 1 and the WP‐SNP index of which is about 1/3 (the ratio of locus ‘a’ in WP is 2a/ [A + A + 2(A + a)] = 1/3).

### Genetic complementation of *ospid‐4*

The vector for complementation test, *pCAMBIA1300‐OsPID*, consisting of the entire CDS, the upstream 5010 bp and the downstream 2002 bp DNA sequence of *OsPID*, was constructed using Gibson Assembly (Gibson *et al.*, 2009). In addition, another two complementary vectors, *pCAMBIA1300‐OsPID‐GFP* and *pCAMBIA1300‐GFP*‐*OsPID*, were constructed on the basis of *pCAMBIA1300‐OsPID*. Three complementary vectors were transformed into *Agrobacterium tumefaciens* (*A. tumefaciens*) strain EHA105 and introduced to the homozygous *ospid‐4* callus from the progeny of heterozygous (*OsPID+/*−) plants, respectively. All the genetic transformations of rice were performed by Biogle Biotechnology (Changzhou, China).

The *AtPID::OsPID* plasmid was transformed into *A. tumefaciens* GV3101, then transformed into two heterozygous (*PID*+/−) lines harbouring the mutation of *pid‐1* and *pid‐3* using the floral dip method (Clough and Bent, 1998).

### qRT‐PCR analysis

Total RNA was extracted from different tissues (including the roots, stems and leaves of rice seedlings (2 weeks) in the vegetative growth period, and panicles of 0.5, 2, 4, 8, 16 and 22.5 cm in the reproductive growth period, and panicles of 1, 5, 10 and 25 days after fertilization) using the RNeasy Plant Mini Kit (cat. nos. 74903 and 74904) according to the manufacturer’s instructions. 2 μg of total RNA was reverse‐transcribed into first‐strand cDNA with FastQuant RT Super Mix (#KR108; Tiangen, Beijing, China). qRT‐PCR was performed with a C1000 Touch^TM^ Thermal Cycler (#785BR05170, Bio‐Rad, Hercules, CA) and SuperReal PreMix (SYBR Green) kit (#FP204‐02; TIANGEN). *OsActin1* was used as a reference gene. Three biological replicates were performed. The primers used here were listed in Table S10.

### Construction of *OsPID::OsPID‐GUS* and β‐glucuronidase (GUS) staining

*OsPID::OsPID‐GUS*, consisting of the entire CDS, the upstream 5010 bp DNA sequence of *OsPID* and *GUS* sequence, was constructed using Gibson Assembly (Gibson *et al.*, 2009). The primers used here were listed in Table S10. This vector was transformed into *A. tumefaciens* strain EHA105 and then transformed into ZH11 callus. The florets, spikelets and roots were removed from transgenic plants and stained with *GUS* staining solution as described previously (Chen *et al.*, 2007). Images were captured using Axioskop 2 Plus microscope under UV light.

### *In situ* hybridization

The *in situ* hybridization experiment was performed according to the previous protocol (Kouchi and Hata, 1993) with minor modification. Young panicles were dissected and fixed in solution [4% (w/v) paraformaldehyde and 0.25% glutaraldehyde in 0.1 M sodium phosphate buffer (pH 7.4)] overnight at 4 °C. Then the samples were dehydrated through conventional ethanol series, infiltrated with xylene and embedded in Paraplast Plus (Sigma, St Louis, MO). The sections (8 μm thick) were made with a Leica RM2265 rotary microtome. The *OsPID* fragment (1–270 bp starting from the ATG start codon) was amplified then transcribed in vitro using DIG RNA Labeling T7/SP6 Kit (Roche, Basel, Switzerland) for antisense or sense probes. The primers used here were listed in Table S10.

### Bioinformatics analysis

The protein sequence OsPID was analysed in the Phytozome (https://phytozome.jgi.doe.gov/pz/portal.html) database, and the top 20 homologous proteins for alignment were selected to construct phylogenetic tree with MEGA5 (Tamura *et al.*, 2011) by neighbour‐joining (NJ) method. The conserved domains of OsPID and homologs were identified using the Pfam database (http://pfam.sanger.ac.uk/). Multiple sequence alignment was performed with MEGA5 and showed on a website (http://www.bio-soft.net/sms/index.html).

### Subcellular localization of OsPID

The coding sequence of *OsPID* was cloned into the *pSAT6‐EYFP‐C1* and *pSAT6‐EYFP‐N1* vectors (Tzfira *et al.*, 2005) to generate GFP‐OsPID and OsPID‐GFP protein fusion constructs for transfection into rice protoplasts. The primers used here were listed in Table S10. Subsequent transfection was carried out as described previously (Zhang *et al.*, 2011b).

The promoters (the upstream 5010 bp DNA sequence of *OsPID*) of *pCAMBIA1300‐OsPID‐GFP* and *pCAMBIA1300‐GFP‐OsPID* plasmids were replaced by ubiquitin promoter of maize to generate *pUbi*::*GFP*‐*OsPID* and *pUbi*::*OsPID*‐*GFP* plasmids. Two plasmids were transformed into *A. tumefaciens* strain EHA105 and then ZH11 calli. The localization of OsPID in roots from transgenic plants was observed using confocal laser scanning microscope (Zeiss LSM 510 Meta).

### Y2H assay

Y2H assay was performed according to the YEASTMAKER Yeast Transformation System 2 User Manual (Clontech). The cDNA of *OsPID* was cloned into *pGADT7*, and the cDNAs of *LAX1*, *OsMADS16*, *LOC_Os02g27030*, *LOC_Os04g31910* and *LOC_Os06g12580* were cloned into *pGBKT7* with Gibson Assembly, respectively. The primers used here were listed in Table S10. The paired bait and prey plasmids were co‐transformed into yeast stain AH109. Protein interactions were tested under the growth conditions on synthetic dropout medium without Trp, Leu, Ade and His at 28°C.

### BiFC

The cDNA from *OsPID* was subcloned into *SCYNE(R)* vector, and the cDNA of *OsPIN1a*, *OsPIN1b* and *OsMADS16* were subcloned into SCYCE vector, respectively. The primers were listed in Table S10. *A. tumefaciens* GV3101 carrying SCYNE(R)::OsPID/ SCYCE::OsPIN1a, SCYNE(R)::OsPID/ SCYCE::OsPIN1b and SCYNE(R)::OsPID/ SCYCE::OsMADS16 plasmids were used together with the p19 strain for infiltration of 4‐ to 6‐week‐old tobacco leaves. The experiment was performed according to previous protocol (Waadt *et al.*, 2008).

### *In vitro* pull‐down assay

The cDNA of *OsPID* was subcloned into *pET‐28a* vector for fusing the HIS‐tag, and the cDNA of *OsMADS16*, *OsPIN1a* and *OsPIN1b* were subcloned into *pGEX4T‐2* for fusing the GST‐tag, respectively. The primers were listed in Table S10. Vectors were transformed into *Escherichia coli* strain BL21, and the expression of the fusion proteins was induced with 0.5 mM IPTG at 16°C for 12 h. The pull‐down assay was performed according to previous protocol (Miernyk and Thelen, 2008). The original Western blot images were shown in Figure S6.

### *In vivo* Co‐IP assay

The CDS of *OsPID* was inserted into *pBluescript II SK (pBSK)(+)‐35S‐N‐3 × FLAG* vector for fusing the FLAG‐tag, and the CDS of *GST*, *OsMADS16*, *OsPIN1a* and *OsPIN1b* were inserted into *pBSK(+)‐35S‐C‐GFP* for fusing the GFP tag, respectively. Vectors were co‐transformed into rice protoplasts. The primers were listed in Table S10. The Co‐IP assay was carried out according to the previous protocol (Yang *et al.*, 2014). The original Western blot figures were shown in Figure S7.

## Conflict of Interest

The authors declare that there is no conflict of interest.

## Author contributions

WCY, DQS, ZST and HMW designed the experiments; HMW and DJX performed the experiments; HMW and WCY prepared the manuscript.

## Supporting information


**Figure S1** Comparison of pollen sacs and embryo sacs from WT and *ospid‐4*.
**Figure S2** Amino acid alignment, diagram of complementaion construct and phenotypic comparison of different lines.
**Figure S3** Alignment of OsPID homologs from different species.
**Figure S4** Phenotypic analysis of *AtPID::OsPID* transgenic plants.
**Figure S5** Subcellular localization of OsPID and panicle comparison.
**Figure S6** The original Western blot images of in vitro pull‐down assay.
**Figure S7** The original Western blot figures of *in vivo* Co‐IP analysis.
**Table S1** Statistics of hull types of *ospid‐4* and *ospid‐cr*.
**Table S2** The segregation of F_2_ population.
**Table S3** Statistics of stamen numbers in WT and *ospid‐4* florets.
**Table S4** Phenotypic statistics of pollen sacs in *ospid‐4* and *ospid‐cr* florets.
**Table S5** Statistics of pistil types of *ospid‐4* and *ospid‐cr*.
**Table S6** Statistics of double‐ovule pistil of *ospid‐4*.
**Table S7** Statistics of stamen numbers in WT and *ospid‐cr*.
**Table S8** Statistics of pistil types and hull types of transgenic plants.
**Table S9** Statistics of the number of stamens and pollen sac types of transgenic plants.
**Table S10** The primers used in this study.
