# Supplementary material for: PINOID regulates floral organ development by modulating auxin transport and interacts with MADS16 in rice
Source: Plant Biotechnol J. 2020 Feb 4;18(8):1778–95. doi: 10.1111/pbi.13340 (PMC7336374; doi:10.1111/pbi.13340)
Supplement: Supplementary file 1 — Figure S1 Comparison of pollen sacs and embryo sacs from WT and ospid‐4. Figure S2 Amino acid alignment, diagram of complementaion construct and phenotypic comparison of different lines. Figure S3 Alignment of OsPID homologs from different species. Figure S4 Phenotypic analysis of AtPID::OsPID transgenic plants. Figure S5 Subcellular localization of OsPID and panicle comparison. Figure S6 The original Western blot images of in vitro pull‐down assay. Figure S7 The original Western blot figures of in vivo Co‐IP analysis. Table S1 Statistics of hull types of ospid‐4 and ospid‐cr. Table S2 The segregation of F2 population. Table S3 Statistics of stamen numbers in WT and ospid‐4 florets. Table S4 Phenotypic statistics of pollen sacs in ospid‐4 and ospid‐cr florets. Table S5 Statistics of pistil types of ospid‐4 and ospid‐cr. Table S6 Statistics of double‐ovule pistil of ospid‐4. Table S7 Statistics of stamen numbers in WT and ospid‐cr. Table S8 Statistics of pistil types and hull types of transgenic plants. Table S9 Statistics of the number of stamens and pollen sac types of transgenic plants. Table S10 The primers used in this study. [file PBI-18-1778-s001.docx]

***Plant Biotechnology Journal* Supporting Information**

Article title: **PINOID regulates floral organ development by modulating auxin transport and interacts with MADS16 in rice**

Authors: Hua-Mao Wu^1, 2^, Dong-Jiang Xie^1, 2^, Zuo-Shun Tang^1, 2^, Dong-Qiao Shi^1, 2^ and Wei-Cai Yang^1, 2, #^

Running title: **OsPINOID Regulates Stamen and Stigma Development**

The following Supporting Information is available for this article:

**Fig. S1** Comparison of pollen sacs and embryo sacs from WT and *ospid-4*.

**Fig. S2** Amino acid alignment, diagram of complementation construct and phenotypic comparison of different lines.

**Fig. S3** Alignment of OsPID homologs from different species.

**Fig. S4** Phenotypic analysis of *AtPID::OsPID* transgenic plants.

**Fig. S5** Subcellular localization of OsPID and panicle comparison.

**Fig. S6** The original Western blot images of *in vitro* pull-down assay.

**Fig. S7** The original Western blot figures of *in vivo* Co-IP analysis.

**Table S1** Statistics of hull types of *ospid-4* and *ospid-cr*.

**Table S2** The segregation of F_2_ population.

**Table S3** Statistics of stamen numbers in WT and *ospid-4* florets.

**Table S4** Phenotypic statistics of pollen sacs in *ospid-4* and *ospid-cr* florets.

**Table S5** Statistics of pistil types of *ospid-4* and *ospid-cr*.

**Table S6** Statistics of double-ovule pistil of *ospid-4*.

**Table S7** Statistics of stamen numbers in WT and *ospid-cr*.

**Table S8** Statistics of pistil types and hull types of transgenic plants.

**Table S9** Statistics of the number of stamens and pollen sac types of transgenic plants.

**Table S10** The primers used in this study.

**Fig. S1** Comparison of pollen sacs and embryo sacs from WT and *ospid-4*. (a) The types of pollen sacs from WT and *ospid-4*. Left-most, normal stamens with TLP and TSSP in WT florets. Anthers with TLP and TSSP (I), TLP and two short pollen sacs (II), TLP and one short pollen sacs (III), only TLP (IV), and two adhesive anthers (V) in *ospid-4* florets. Bar, 0.5 mm. (b) Comparison of embryo sacs (Whole mount clearing) from WT (left) and *ospid-4* (right). AN, antipodal cell nucleus; PN, polar nucleus; SN, synergid nucleus; EN, egg nucleus. Bars, 20 μm.

**
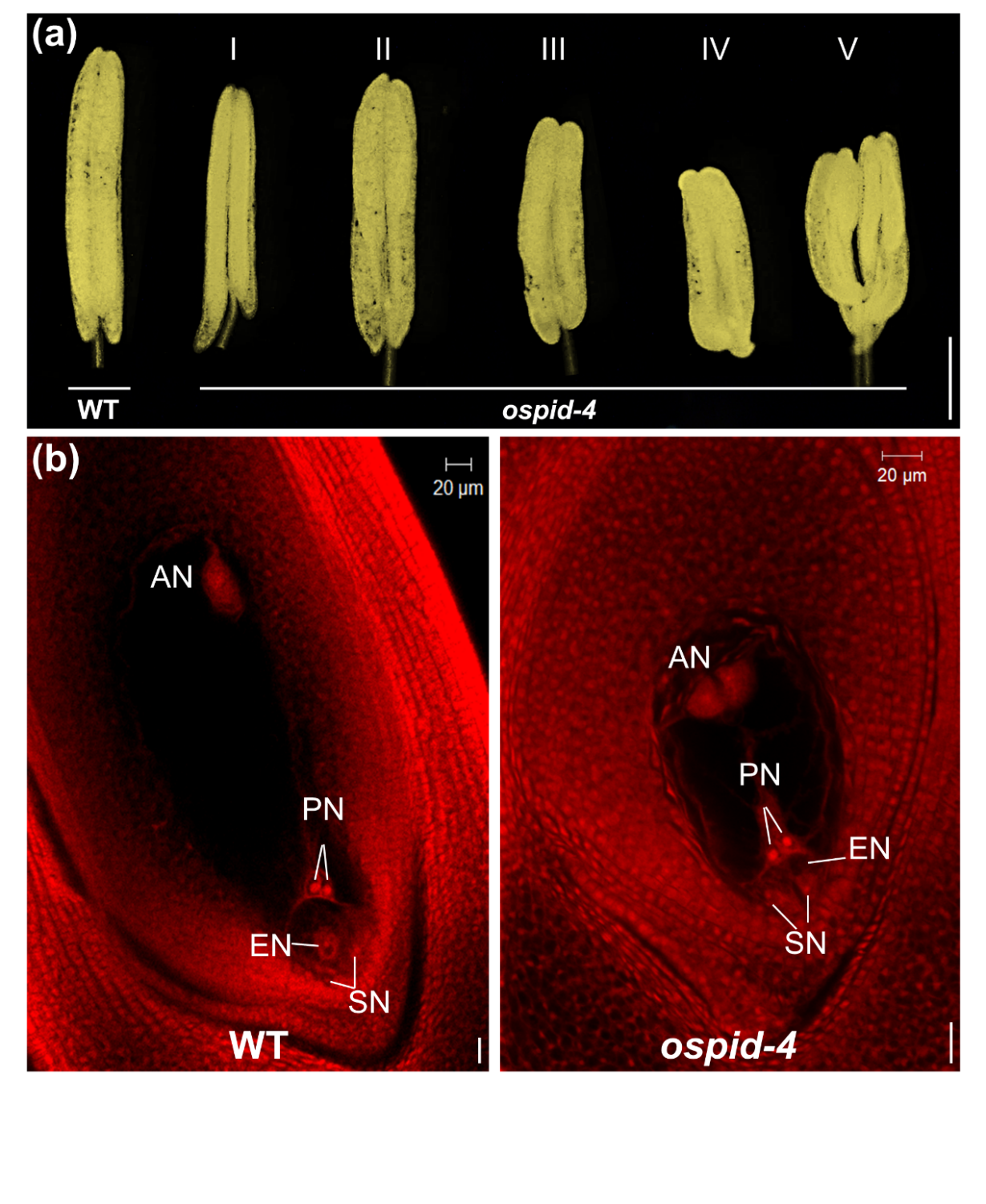
**

**Fig. S2** Amino acid alignment, diagram of complementaion construct and phenotypic comparison of different lines. (a) Amino acid alignment of OsPID, *ospid-4* and *ospid-cr* proteins. *ospid-4* contained an amino acid substitution (249^Leu>Phe^) labeling with a red frame, *ospid-cr* harbored a deletion of base A256 in the CDS region, which leads to a premature stop codon (106^CTA>TAG^) labeling with a blue frame. Similar amino acids among them are shaded in gray. (b) Diagram of *OsPID* sequence of genetic complementation. Gray frame represents upstream or downstream sequence of *ORF2*, blue frame shows coding region. (c) Phenotypic comparison of WT, *ospid-4*, *ospid-cr* and L74-1-4. Bar, 10 cm.

**
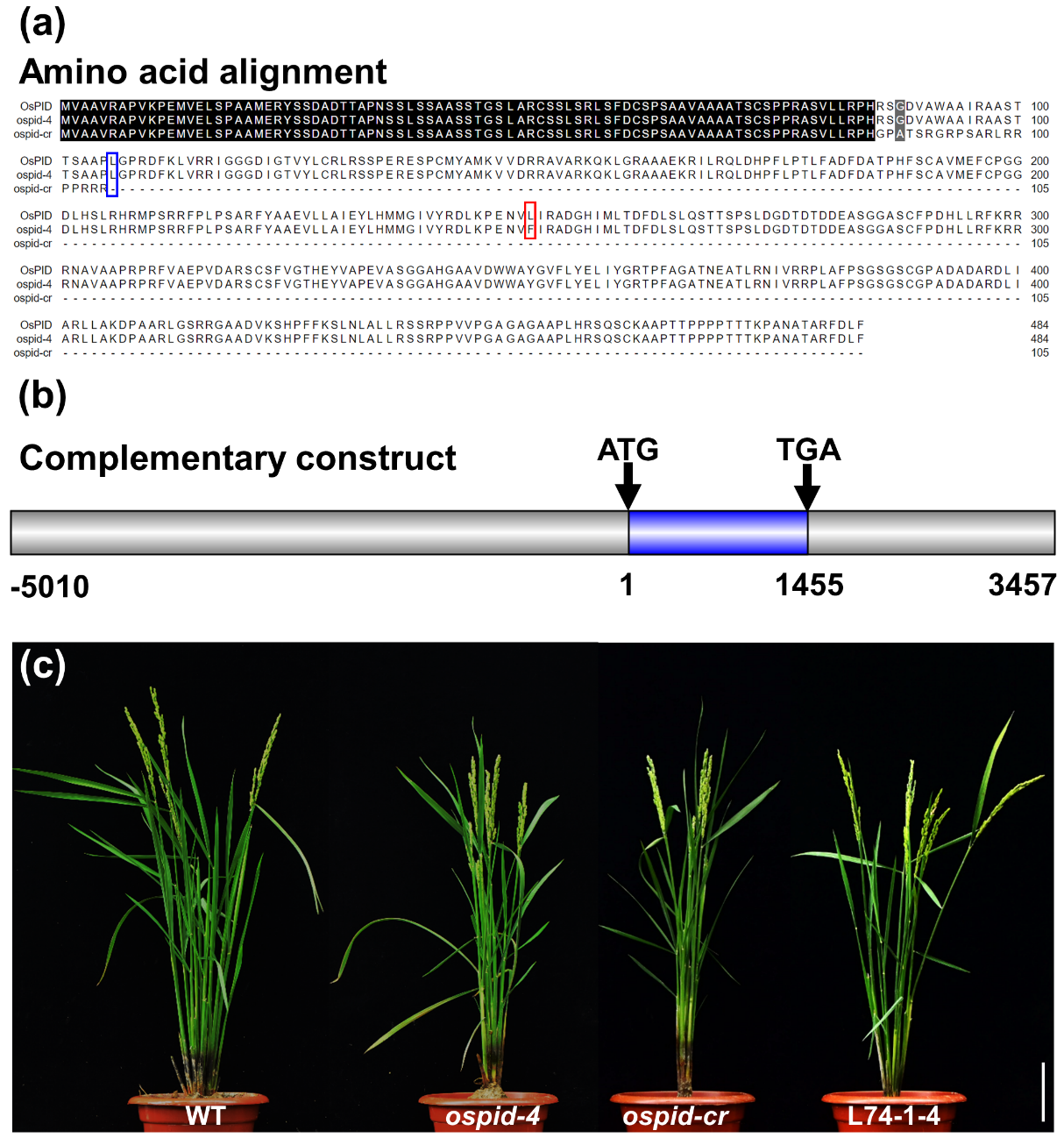
**

**Fig. S3** Alignment of OsPID homologs from different species. Pkinase domains are labeled with red line and the mutation of amino acids is highlighted in blue frame.

**
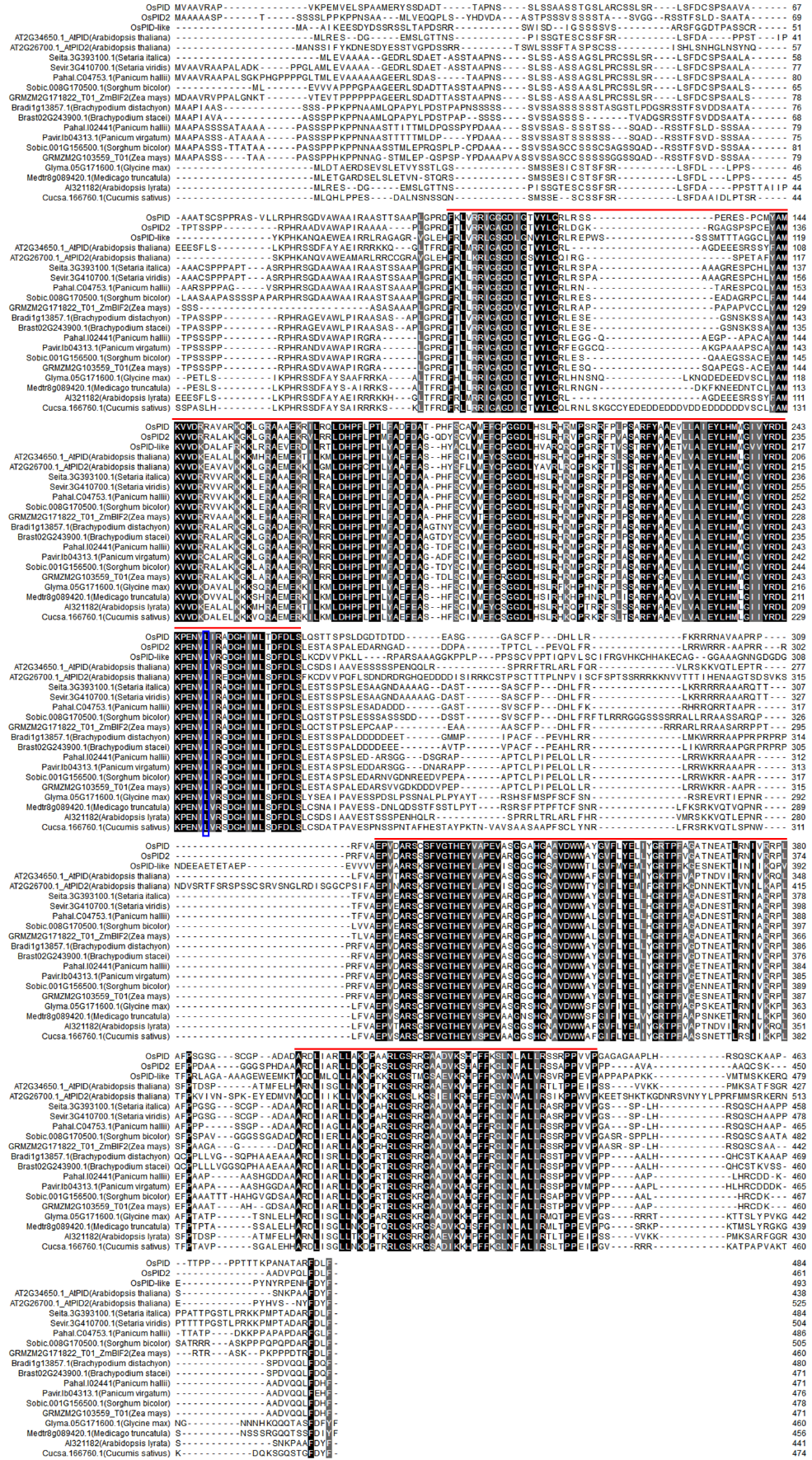
**

**Fig. S4** Phenotypic analysis of *AtPID::OsPID* transgenic plants. (a) Comparison of L*er*, *pid-1* and L2-6 in flowering stage. Bar, 2 cm. (b,c) Comparison of the number of petals (b) and silique morphology (c) in L*er*, *pid-1* and L2-6. Bars, 2 mm. (d) Comparison of Col-3, *pid-3* and L4-5 in flowering stage. Bar, 2 cm. (e,f) Comparison of the number of petals (e) and silique morphology (f) in Col-3, *pid-3* and L4-5. Bars, 2 mm.


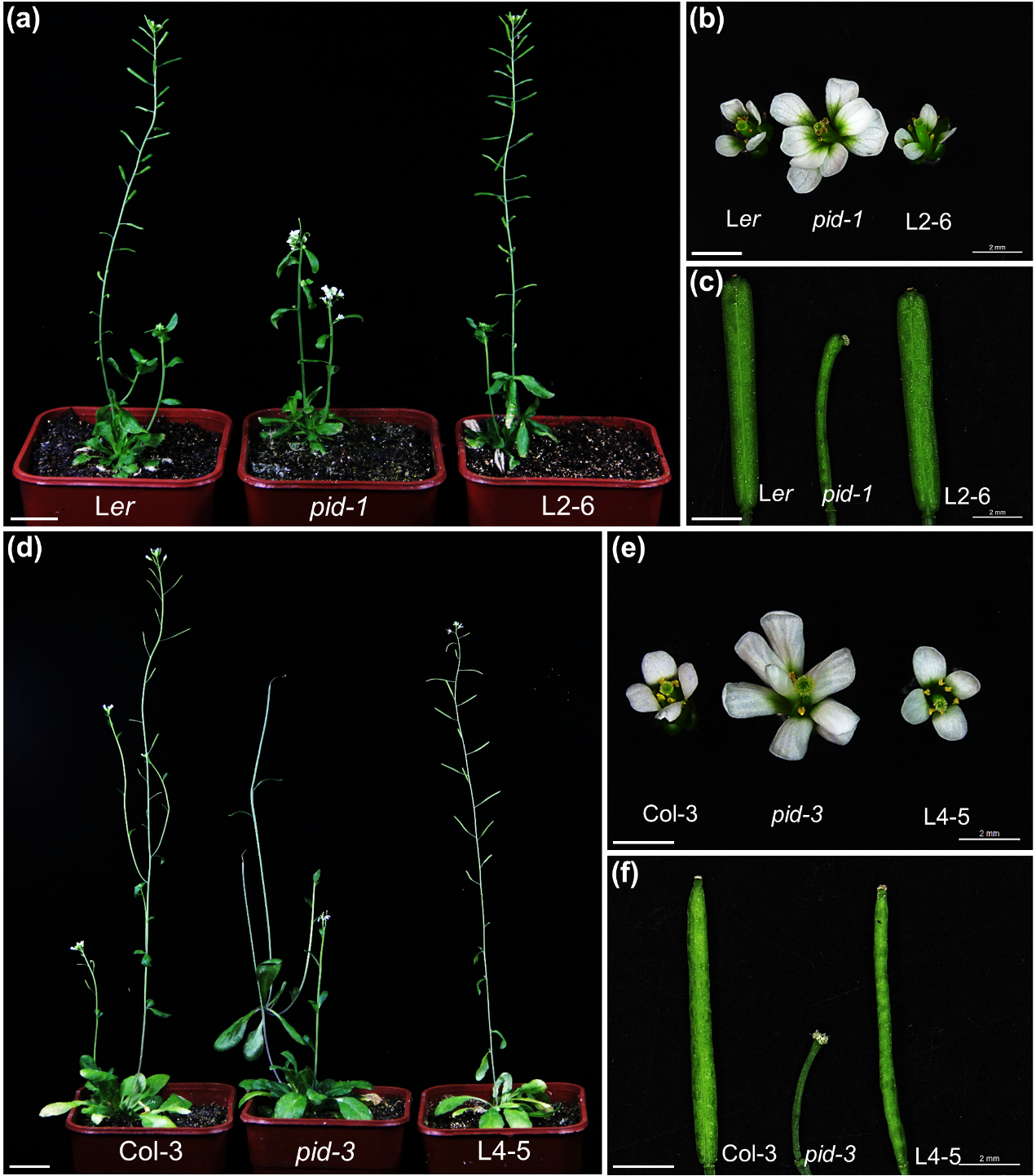


**Fig. S5** Subcellular localization of OsPID and panicle comparison. (a,b) Subcellular localization of OsPID in rice roots expressing *pUBI::GFP-OsPID* (a) and *pUBI::OsPID-GFP* (b) vectors. pUBI::GFP-OsPID is co-localized with FM4-64 on the plasma membrane (a), pUBI::OsPID-GFP is co-localized with DAPI in the nucleus, and FM4-64 on the plasma membrane (b). Bars, 10 μm. (c,d) Panicle comparison of WT, *ospid-4* (c) and *ospid-cr2* (d). The *ospid-cr2* panicle, in red box, has fewer panicle branches and florets compared to WT (d). Bars, 5 cm.

**
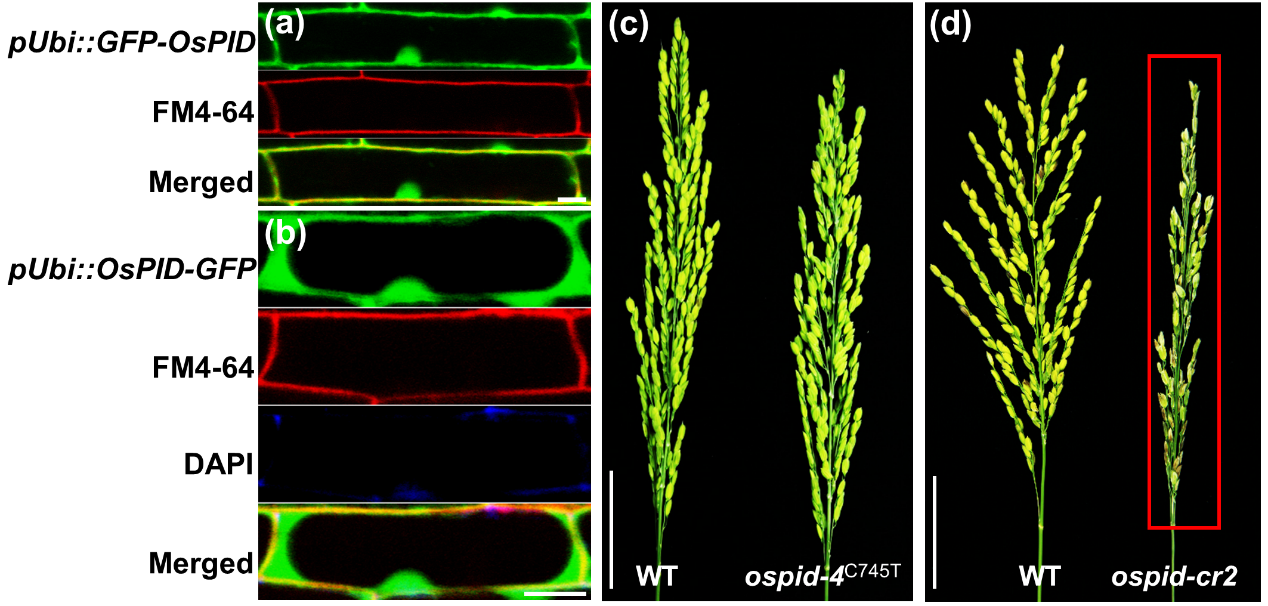
**

**Fig. S6** The original Western blot images of *in vitro* pull-down assay. *In vitro* pull-down assay verified the interactions between OsPID and OsMADS16 (left column), OsPIN1a (middle column), and OsPIN1b (right column). PD, Pull-down. Anti-HIS and anti-GST antibodies were used to detect HIS and GST peptides, respectively.

**
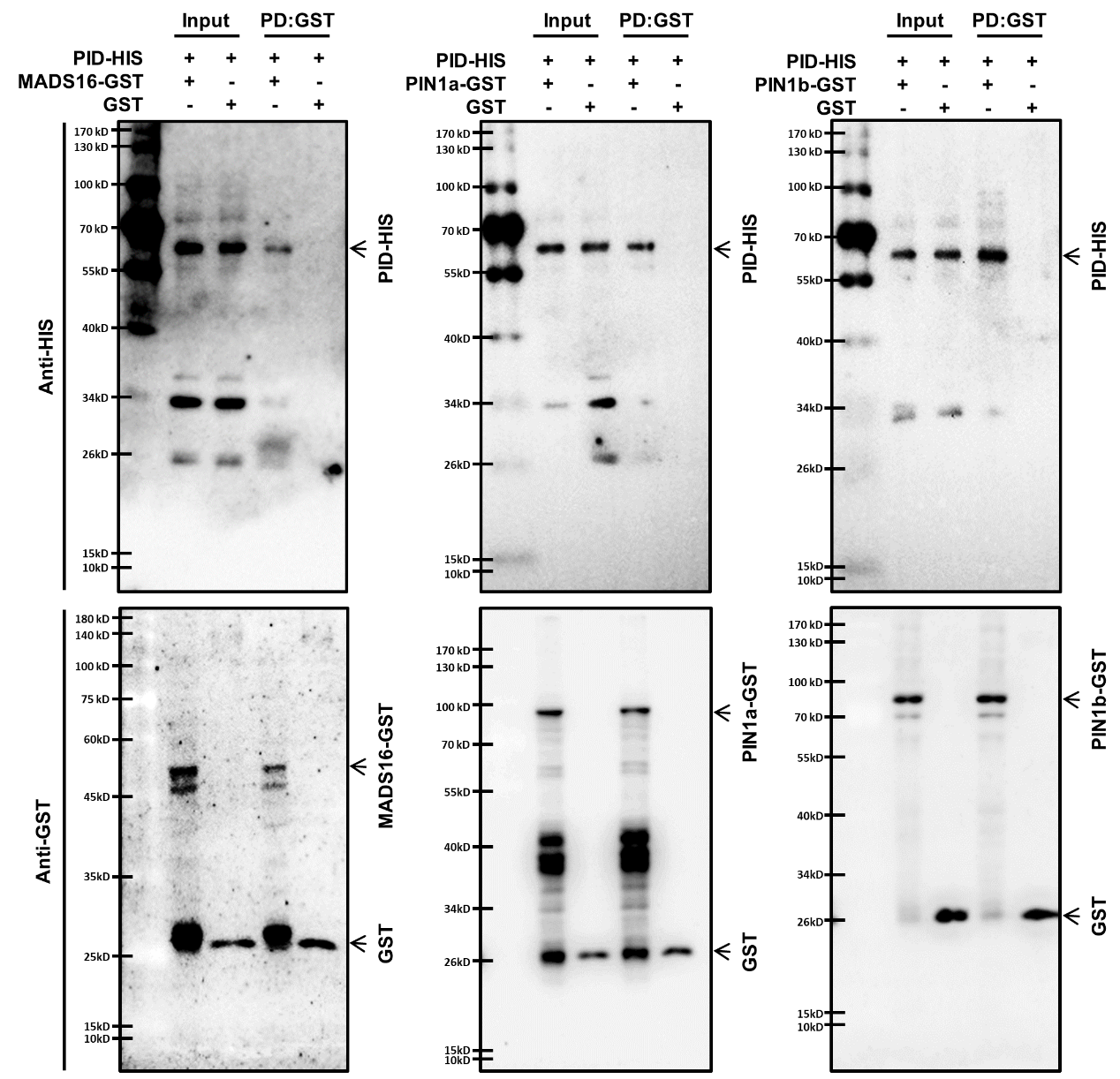
**

**Fig. S7** The original Western blot figures of *in vivo* Co-IP analysis. *In vivo* Co-IP analysis validates the interactions between OsPID and OsMADS16 (left column), OsPIN1a (middle column), and OsPIN1b (right column). IP, co-immunoprecipitation. Anti-FLAG and anti-GFP antibodies were used to detect FLAG and GFP peptides, respectively. GFP and FLAG-GST were used as negative controls.

**
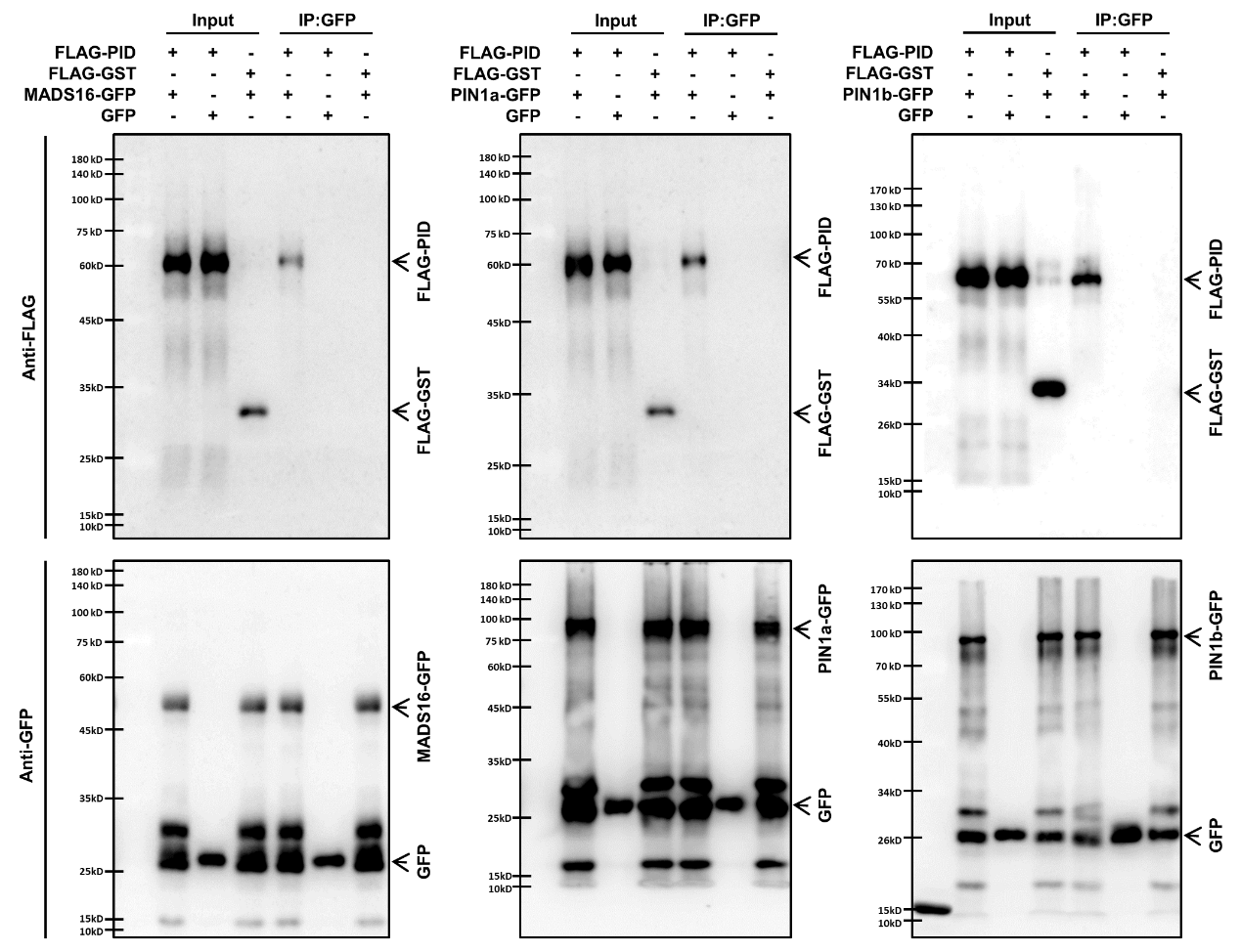
**

**Table S1** Statistics of hull types of *ospid-4* and *ospid-cr*

| Hull types | Total | I | II | III | IV | V |
| --- | --- | --- | --- | --- | --- | --- |
| NO. (*ospid-4*) | 406 | 307 | 70 | 5 | 16 | 8 |
| Ratio (%) | 100 | 75.6 | 17.2 | 1.2 | 4 | 2 |
| NO. (*ospid-cr*) | 308 | 241 | 59 | 6 | 1 | 1 |
| Ratio (%) | 100 | 78.2 | 19.2 | 1.9 | 0.3 | 0.3 |

**Table S2** The segregation of F_2_ population

| Mutant | NO. of WT | NO. of mutant | Total plants | χ^2^ (3 : 1) | χ^2^_0.05_ (1) | *P* value |
| --- | --- | --- | --- | --- | --- | --- |
| *ospid-4* | 164 | 44 | 208 | 1.44 (NS) | 3.84 | > 0.05 |

NS, Nonsignificant.

**Table S3** Statistics of stamen numbers in WT and *ospid-4* florets

| Plants | Stigma types | | | | | | Stamens | |
| --- | --- | --- | --- | --- | --- | --- | --- | --- |
|  | With stigma | | | Without stigma | | | Total | Ratio |
|  | Double stigmas with more stigma hair | Double stigmas with less stigma hair | Single stigma with less stigma hair | Short-double styles without stigma | Short-single style without stigma | No style |  |  |
| WT | 211 | 0 | 0 | 0 | 0 | 0 | Total | Ratio (n = 211) |
| 6 stamens | 211 | 0 | 0 | 0 | 0 | 0 | 211 | 100% |
| *ospid-4* (T130-36) | 0 | 37 | 16 | 41 | 57 | 50 | Total | Ratio (n = 201) |
| 6 stamens | 0 | 35 | 11 | 39 | 47 | 44 | 176 | 87.6% |
| 7stamens | 0 | 2 | 3 | 1 | 4 | 3 | 13 | 6.5% |
| 8stamens | 0 | 0 | 2 | 1 | 6 | 2 | 11 | 5.5% |
| 9stamens | 0 | 0 | 0 | 0 | 0 | 1 | 1 | 0.5% |
| *ospid-4* (T136-1-8) | 0 | 34 | 17 | 38 | 53 | 58 | Total | Ratio (n = 200) |
| 6 stamens | 0 | 33 | 15 | 33 | 46 | 49 | 176 | 88.0% |
| 7stamens | 0 | 1 | 1 | 5 | 3 | 5 | 15 | 7.5% |
| 8stamens | 0 | 0 | 1 | 0 | 4 | 4 | 9 | 4.5% |
| *ospid-4* (T136-2-4) | 0 | 32 | 9 | 74 | 50 | 37 | Total | Ratio (n = 202) |
| 6 stamens | 0 | 30 | 6 | 65 | 36 | 29 | 166 | 82.2% |
| 7stamens | 0 | 2 | 1 | 6 | 8 | 6 | 23 | 11.4% |
| 8stamens | 0 | 0 | 2 | 2 | 6 | 2 | 12 | 5.9% |
| 9stamens | 0 | 0 | 0 | 1 | 0 | 0 | 1 | 0.5% |
| **Average ratio of *ospid-4* florets had more than six stamens (n = 603)** | | | | | | | | **14.1%** |

**Table S4** Phenotypic statistics of pollen sacs in *ospid-4* and *ospid-cr* florets

| Plants | Anthers types | | | | |
| --- | --- | --- | --- | --- | --- |
|  | Four pollen sacs and normal length (I) | Two normal pollen sacs and two short pollen sacs  (II) | Two normal pollen sacs and one short pollen sac  (III) | Two curved pollen sacs (IV) | Two adhesive anthers (V) |
| WT | 1266 | 0 | 0 | 0 | 0 |
| Ratio (n = 1266) | 100% | 0% | 0% | 0% | 0% |
| *ospid-4* | 0 | 202 | 665 | 2780 | 44 |
| Ratio (n = 3692) | 0.03% | 5.47% | 18.01% | 75.30% | 1.19% |
| *ospid-cr* | 0 | 521 | 633 | 841 | 23 |
| Ratio (n = 2018) | 0% | 25.8% | 31.4% | 41.7% | 1.1% |

**Table S5** Statistics of pistil types of *ospid-4* and *ospid-cr*

| Plants | Stigma types | | | | | |
| --- | --- | --- | --- | --- | --- | --- |
|  | With stigma | | | Without stigma | | |
|  | Double stigmas with more stigma hair (WT) | Double stigmas with less stigma hair (I) | Single stigma with less stigma hair  (II) | Short-double styles without stigma (III) | Short-single style without stigma (IV) | No style (V) |
| WT | 211 | 0 | 0 | 0 | 0 | 0 |
| Ratio (n = 211) | 100% | 0% | 0% | 0% | 0% | 0% |
| *ospid-4* | 0 | 101 | 41 | 155 | 161 | 145 |
| Ratio (n = 603) | 0% | 16.7% | 6.8% | 25.6% | 26.6% | 24.0% |
| *ospid-cr* | 0 | 3 | 20 | 75 | 139 | 71 |
| Ratio (n = 308) | 0% | 1.0% | 6.5% | 24.4% | 45.1% | 23.1% |

**Table S6** Statistics of double-ovule pistil of *ospid-4*

| Plants | Stigma types | | | | | | Ovules | |
| --- | --- | --- | --- | --- | --- | --- | --- | --- |
|  | With stigma | | | Without stigma | | | Total | Ratio |
|  | Double stigmas with more stigma hair | Double stigmas with less stigma hair | Single stigma with less stigma hair | Short-double styles without stigma | Short-single style Without stigma | No style |  |  |
| WT | 140 | 0 | 0 | 0 | 0 | 0 | Total | Ratio (%) (n = 140) |
| Single ovule | 140 | 0 | 0 | 0 | 0 | 0 | 140 | 100.00 |
| Double-ovule | 0 | 0 | 0 | 0 | 0 | 0 | 0 | 0.00 |
| *ospid-4* (T130-36) | 0 | 33 | 150 | 26 | 83 | 180 | Total | Ratio (%) (n = 472) |
| Single ovule | 0 | 32 | 147 | 26 | 78 | 176 | 459 | 97.25 |
| Double-ovule | 0 | 1 | 3 | 0 | 5 | 4 | 13 | 2.75 |
| *ospid-4* (T136-1-8) | 0 | 5 | 25 | 8 | 38 | 69 | Total | Ratio (%) (n = 145) |
| Single ovule | 0 | 5 | 24 | 4 | 35 | 68 | 136 | 93.79 |
| Double-ovule | 0 | 0 | 1 | 4 | 3 | 1 | 9 | 6.21 |
| *ospid-4* (T136-2-4) | 0 | 3 | 28 | 11 | 22 | 56 | Total | Ratio (%) (n = 120) |
| Single ovule | 0 | 3 | 27 | 10 | 21 | 55 | 116 | 96.67 |
| Double-ovule | 0 | 0 | 1 | 1 | 1 | 1 | 4 | 3.33 |
| **Average ratio of *ospid-4* (n = 737)** | | | | | | | | **4.10%** |

**Table S7** Statistics of stamen numbers in WT and *ospid-cr*

| Plants | Stigma types | | | | | | | Stamens | |
| --- | --- | --- | --- | --- | --- | --- | --- | --- | --- |
|  | With stigma | | | Without stigma | | | | Total | Ratio |
|  | Double stigmas with more stigma hair | Double stigma with less stigma hair | Single stigma with less stigma hair | | Short-double styles without stigma | Short-single style without stigma | No style |  |  |
|  |  |  |  |  |  |  |  |  |  |
| WT | 211 | 0 | 0 | | 0 | 0 | 0 | Total | Ratio  (n = 211) |
| 6 stamens | 211 | 0 | 0 | | 0 | 0 | 0 | 211 | 100% |
| *ospid-cr* | 0 | 1 | 20 | | 141 | 75 | 71 | Total | Ratio  (n = 308) |
| 6 stamens | 0 | 0 | 16 | | 99 | 50 | 45 | 210 | 68.2% |
| 7stamens | 0 | 0 | 3 | | 23 | 15 | 14 | 55 | 17.9% |
| 8stamens | 0 | 1 | 1 | | 18 | 10 | 9 | 39 | 12.7% |
| 9stamens | 0 | 0 | 0 | | 0 | 0 | 3 | 3 | 1.0% |
| 10stamens | 0 | 0 | 0 | | 1 | 0 | 0 | 1 | 0.3% |
| **The ratio of *ospid-cr* florets had more than 6 stamens (n = 308)** | | | | | | | | | **31.8%** |

**Table S8** Statistics of pistil types and hull types of transgenic plants

| Plants | Hull type | Stigma types | | | | | | | |
| --- | --- | --- | --- | --- | --- | --- | --- | --- | --- |
|  |  | With stigma | | | | Without stigma | | | |
|  |  | Double stigmas with more stigma hair | Three stigmas with more stigma hair | Double stigmas with less stigma hair | Single stigma with less stigma hair | | Short-double styles without  stigma | Short-single style without  stigma | No style |
| WT | Normal | 140 | 0 | 0 | 0 | | 0 | 0 | 0 |
| Ratio  (n = 140) | 100% | 100% | 0% | 0% | 0% | | 0% | 0% | 0% |
| T74-1-4 | Normal | 191 | 3 | 0 | 0 | | 0 | 0 | 0 |
| Ratio  (n = 194) | 100% | 98.5% | 1.5% | 0% | 0% | | 0% | 0% | 0% |
| T74-2-4 | Normal | 92 | 1 | 0 | 0 | | 0 | 0 | 0 |
| Ratio  (n = 93) | 100% | 98.9% | 1.1% | 0% | 0% | | 0% | 0% | 0% |
| T74-3-1 | Normal | 92 | 4 | 0 | 0 | | 0 | 0 | 0 |
| Ratio  (n = 96) | 100% | 95.8% | 4.2% | 0% | 0% | | 0% | 0% | 0% |

**Table S9** Statistics of the number of stamens and pollen sac types of transgenic plants

| Plants | Stamen number | Anthers types | | | | |
| --- | --- | --- | --- | --- | --- | --- |
|  |  | Four pollen sacs and  normal length | Two normal pollen sacs  and two short pollen sacs | Two normal pollen sacs  and one short pollen sac | Two bow pollen sacs | Two adhesive anthers |
| WT | 6 | 840 | 0 | 0 | 0 | 0 |
| Ratio (n =840) | 100% | 100% | 0% | 0% | 0% | 0% |
| T74-1-4 | 6 | 1095 | 69 | 0 | 0 | 0 |
| Ratio (n = 1,164) | 100% | 94.1% | 5.9% | 0.0% | 0.0% | 0.0% |
| T74-2-4 | 6 | 525 | 24 | 9 | 0 | 0 |
| Ratio (n = 558) | 100% | 94.1% | 4.3% | 1.6% | 0.0% | 0.0% |
| T74-3-1 | 6 | 530 | 46 | 0 | 0 | 0 |
| Ratio (n = 576) | 100% | 92.0% | 8.0% | 0.0% | 0.0% | 0.0% |

**Table S10** The primers used in this study

| Primer name | Annotation | Sequences (5'-3') |
| --- | --- | --- |
| *OsPIDMF* | Identification | GCATGTACGCGATGAAGGTG |
| *OsPIDMR* | Identification | GTACGGCCGTAGATGAGCTC |
| *ProOsPID-F* | Complementation | CATGATTACGAATTCGAGCTGACTGGACTGAACTCTGCCA |
| *ProOsPID-R* | Complementation | GCACCGCCGCCACCATGTTAACGAACGTGAGCACGTGACACT |
| *CDS^OsPID^-F* | Complementation | TCACGTGCTCACGTTCGTTAACATGGTGGCGGCGGTGCGCGC |
| *CDS^OsPID^-R* | Complementation | ACGGTCAATATGCTCAAGGCCTGAAGAGGTCGAACCGCGCGG |
| *TerOsPID-F* | Complementation | GCGGTTCGACCTCTTCAGGCCTTGAGCATATTGACCGTTGCT |
| *TerOsPID-R* | Complementation | TAGAGGATCCCCGGGTACCGTGGTCCAAGTTAAAGCTAGA |
| *OsPID-GFP-F* | Complementation | GCGGTTCGACCTCTTCAGGATGGTGAGCAAGGGCGAGGA |
| *OsPID-GFP-R* | Complementation | GCAACGGTCAATATGCTCAAGGTTACTTGTACAGCTCGTCC |
| *GFP-OsPID -F* | Complementation | TCACGTGCTCACGTTCGTTAACATGGTGAGCAAGGGCGAGGA |
| *GFP-OsPID -R* | Complementation | CGCACCGCCGCCACCATGTTTTACTTGTACAGCTCGTCCA |
| *pUbi:: OsPID-GFP-F* | Localization | CAGCTATGACCATGATTACGCTGCAGTGCAGCGTGACCCG |
| *pUbi:: OsPID-GFP-R* | Localization | CGCGCACCGCCGCCACCATGTTCTGCAGAAGTAACACCAAAC |
| *pUbi::GFP-OsPID -R* | Localization | CCTCGCCCTTGCTCACCATGTTCTGCAGAAGTAACACCAAAC |
| *CDS-GUS -F* | GUS-line | CGCGCGGTTCGACCTCTTCAGGATGTTACGTCCTGTAGAA |
| *CDS-GUS- R* | GUS-line | AACGGTCAATATGCTCAAGGTCATTGTTTGCCTCCCTGCTGC |
| *Actin1-F* | qRT-PCR | CGTATGAGCAAGGAGATCAC |
| *Actin1-R* | qRT-PCR | CACATCTGTTGGAAGGTGCT |
| *OsPIDqF* | qRT-PCR | AGCACTCGTCCGAGGGCAAAGAAATAG |
| *OsPIDqR* | qRT-PCR | ACAGCAAGGTGATTAGCAGTGA |
| *Pro^ATP^-F* | AtPID::OsPID | CATGATTACGAATTCGAGCTACTTAAGTGTGTATGCATGTGT |
| *Pro^ATP^-R* | AtPID::OsPID | GCGCGCACCGCCGCCACCATCGCCGGGAAAATCGAAGTTA |
| *CDS^OsPID^-AF* | AtPID::OsPID | TAACTTCGATTTTCCCGGCGATGGTGGCGGCGGTGCGCGC |
| *CDS^OsPID^-AR* | AtPID::OsPID | TAATCTCGACCGTAGAAAACGTTCAGAAGAGGTCGAACCGCG |
| *Ter^ATP^-F* | AtPID::OsPID | CGCGGTTCGACCTCTTCTGAACGTTTTCTACGGTCGAGATTA |
| *Ter^ATP^-R* | AtPID::OsPID | TAGAGGATCCCCGGGTACCGTCTTTTTCAAAGTGTAAACAGT |
| *BD-OsPIDF* | Y2H | GCATATGGCCATGGAGGCCGAATTCATGGTGGCGGCGGTGCGCGCGC |
| *BD-OsPIDR* | Y2H | TCGACGGATCCCCGGGAATTCTCAGAAGAGGTCGAACCGCGCGGT |
| *AD-LAX1F* | Y2H | TATGGCCATGGAGGCCAGTGAATTCATGCATGACCCACGCGGC |
| *AD-LAX1R* | Y2H | ATGCCCACCCGGGTGGAATTCCTAATAAGATCCTTGCGCACCA |
| *AD-OsMADS16F* | Y2H | TATGGCCATGGAGGCCAGTGAATTCATGGGGAGGGGCAAGATCG |
| *AD-OsMADS16R* | Y2H | ATGCCCACCCGGGTGGAATTCTCAACCGAGGCGCAGGTC |
| *AD-LOC_Os02g27030F* | Y2H | TATGGCCATGGAGGCCAGTGAATTCATGGATCACCGCCTCCTCCTC |
| *AD-LOC_Os02g27030R* | Y2H | ATGCCCACCCGGGTGGAATTCCTACTCCTTTGAGGTGTGGA |
| *AD-LOC_Os04g431910F* | Y2H | TATGGCCATGGAGGCCAGTGAATTCATGGCCTCCTCGGCGGCGGCG |
| *AD-LOC_Os04g431910R* | Y2H | ATGCCCACCCGGGTGGAATTCTCACACTGAGCAAGGACTCTG |
| *AD-LOC_Os06g12580F* | Y2H | TATGGCCATGGAGGCCAGTGAATTCATGGCGACCGGGTACTACCGC |
| *AD-LOC_Os06g12580R* | Y2H | ATGCCCACCCGGGTGGAATTCTTAATCATCGTACTCCTGGCG |

**Table S10** The primers used in this study (continued)

| Primer name | Annotation | Sequences (5'-3') |
| --- | --- | --- |
| *OsPIDPF* | *In situ* | TGTAATACGACTCACTATAGGGCGAATGGTGGCGGCGGTGCGCGC |
| *OsPIDPR* | *In situ* | ATTTAGGTGACACTATAGAATACAGACGTCGCCGGACCTGTGCGG |
| *pSAT6-C1-OsPID-F* | Localization | GCTCAAGCTTCGAATTCTGCAGTCGACATGGTGGCGGCGGTGCGCGC |
| *pSAT6-C1-OsPID -R* | Localization | GCGGACTCTAGACTAGGTGGATCCCGGTCAGAAGAGGTCGAACCGCG |
| *pSAT6-OsPID-N1-F* | Localization | TCGAGCTCAAGCTTCGAATTCTGCAGTATGGTGGCGGCGGTGCGCGC |
| *pSAT6-OsPID-N1-R* | Localization | GCCCTTGCTCACCATCAGGATCCCGGGGAAGAGGTCGAACCGCGCGG |
| *SCYNE*(*R*)*::OsPID-F* | BiFC | GGGCCCAGGCCTACTAGTGGATCCATGGTGGCGGCGGTGCGCGC |
| *SCYNE*(*R*)*::OsPID-R* | BiFC | CTACCCGGGAGCGGTACCCTCGAGGTCGACTCAGAAGAGGTCGAACC |
| *SCYCE::OsPIN1a-F* | BiFC | GCTCAGGCCTGGCGCGCCACTAGTGGATCCATGATTACGGCGGCGGAC |
| *SCYCE::OsPIN1a-R* | BiFC | CATCCCGGGAGCGGTACCCTCGAGGTCGACCAGCCCAAGCAAGATGT |
| *SCYCE::OsPIN1b-F* | BiFC | TCAGGCCTGGCGCGCCACTAGTGGATCCATGATCACGGTGGTGGACCT |
| *SCYCE::OsPIN1b-R* | BiFC | CATCCCGGGAGCGGTACCCTCGAGGTCGACGAGCCCCAGCAGTATGT |
| *SCYCE::OsMADS16-F* | BiFC | GCTCAGGCCTGGCGCGCCACTAGTGGATCCATGGGGAGGGGCAAGATCG |
| *SCYCE:: OsMADS16-R* | BiFC | CATCCCGGGAGCGGTACCCTCGAGGTCGACACCGAGGCGCAGGTC |
| *pET-28a-OsPID-F* | Pull-down | CGCGGATCCGAATTCGAGCTCCGTATGGTGGCGGCGGTGCGCGCGC |
| *pET-28a-OsPID-R* | Pull-down | AGTGCGGCCGCAAGCTTGTCGACGGAAGAGGTCGAACCGCGCGG |
| *pGEX4T-2-OsPIN1a-F* | Pull-down | CGTGGATCCCCAGGAATTCCCGGGATGATTACGGCGGCGGAC |
| *pGEX4T-2-OsPIN1a-R* | Pull-down | TCACGATGCGGCCGCTCGAGTCGACAGCCCAAGCAAGATGT |
| *pGEX4T-2-OsPIN1b-F* | Pull-down | CGTGGATCCCCAGGAATTCCCGGGATGATCACGGTGGTGGACCT |
| *pGEX4T-2-OsPIN1b-R* | Pull-down | TCACGATGCGGCCGCTCGAGTCGAGAGCCCCAGCAGTATGT |
| *pGEX4T-2-OsMADS16-F* | Pull-down | CGTGGATCCCCAGGAATTCCCGGGATGGGGAGGGGCAAGATCG |
| *pGEX4T-2-OsMADS16-R* | Pull-down | TCACGATGCGGCCGCTCGAGTCGAACCGAGGCGCAGGTCGT |
| *pBSK-FLAG-OsPID-F* | Co-IP | AAGTGCGGCAAGCTTGGAATTCCTGCAATGGTGGCGGCGGTGCGCGCGC |
| *pBSK-FLAG-OsPID-R* | Co-IP | GCTCTAGAACTAGTGGATCCCCCGGGCGAAGAGGTCGAACCGCGCGG |
| *pBSK-FLAG-GST-F* | Co-IP | AAGTGCGGCAAGCTTGGAATTCCTGCAATGTCCCCTATACTAGGTTATTGGA |
| *pBSK-FLAG-GST-R* | Co-IP | GCTCTAGAACTAGTGGATCCCCCGGGCTTTTGGAGGATGGTCGCCAC |
| *pBSK-OsPIN1a-GFP-F* | Co-IP | ATAAGCTTGATATCGAATTCCTGCAATGATTACGGCGGCGGAC |
| *pBSK-OsPIN1a-GFP-R* | Co-IP | TCTAGAACTAGTGGATCCCCCGGCCAGCCCAAGCAAGATGT |
| *pBSK-OsPIN1b-GFP-F* | Co-IP | ATAAGCTTGATATCGAATTCCTGCAATGATCACGGTGGTGGACCT |
| *pBSK-OsPIN1b-GFP-R* | Co-IP | TCTAGAACTAGTGGATCCCCCGGCGAGCCCCAGCAGTATGT |
| *pBSK-OsMADS16-GFP-F* | Co-IP | ATAAGCTTGATATCGAATTCCTGCAATGCATGACCCACGCGGC |
| *pBSK-OsMADS16-GFP-R* | Co-IP | TCTAGAACTAGTGGATCCCCCGGCACCGAGGCGCAGGTCGT |
